# Realistic simulation of virtual multi-scale, multi-modal patient trajectories using Bayesian networks and sparse auto-encoders

**DOI:** 10.1038/s41598-020-67398-4

**Published:** 2020-07-03

**Authors:** Meemansa Sood, Akrishta Sahay, Reagon Karki, Mohammad Asif Emon, Henri Vrooman, Martin Hofmann-Apitius, Holger Fröhlich

**Affiliations:** 1 Department of Bioinformatics, Fraunhofer Institute for Algorithms and Scientific Computing (SCAI), Schloss Birlinghoven, 53754 Sankt Augustin, Germany; 2 Bonn-Aachen International Center for Information Technology (B-IT), University of Bonn, 53115 Bonn, Germany; 3 Department of Radiology and Medical Informatics, Erasmus MC, University Medical Center Rotterdam, PO Box 2040, 3000 CA Rotterdam, The Netherlands; 4 UCB Biosciences GmbH, Alfred-Nobel Str. 10, 40789 Monheim, Germany

**Keywords:** Drug development, Translational research

## Abstract

Translational research of many disease areas requires a longitudinal understanding of disease development and progression across all biologically relevant scales. Several corresponding studies are now available. However, to compile a comprehensive picture of a specific disease, multiple studies need to be analyzed and compared. A large number of clinical studies is nowadays conducted in the context of drug development in pharmaceutical research. However, legal and ethical constraints typically do not allow for sharing sensitive patient data. In consequence there exist data “silos”, which slow down the overall scientific progress in translational research. In this paper, we suggest the idea of a virtual cohort (VC) to address this limitation. Our key idea is to describe a longitudinal patient cohort with the help of a generative statistical model, namely a modular Bayesian Network, in which individual modules are represented as sparse autoencoder networks. We show that with the help of such a model we can simulate subjects that are highly similar to real ones. Our approach allows for incorporating arbitrary multi-scale, multi-modal data without making specific distribution assumptions. Moreover, we demonstrate the possibility to simulate interventions (e.g. via a treatment) in the VC. Overall, our proposed approach opens the possibility to build sufficiently realistic VCs for multiple disease areas in the future.

## Introduction

Translational research of many disease areas requires a longitudinal understanding of disease development and progression across all biologically relevant scales. Examples of corresponding observational clinical studies include the Alzheimer’s Disease Neuroimaging Initiative (ADNI) (https://adni.loni.usc.edu/) (omics, neuro-imaging, longitudinal clinical data), the Parkinson’s Progression Markers Initiative (PPMI) (https://www.ppmi-info.org/) (omics, neuro-imaging, longitudinal clinical data), the All-of-us cohort (https://allofus.nih.gov/) (omics, behavioral, electronic medical records, environmental data) and the GENIE project (https://www.aacr.org/Research/Research/Pages/aacr-project-genie.asp) (genomic and longitudinal real-world clinical data). These studies provide unique opportunities to obtain an increasingly holistic view of a patient’s health trajectory and allow e.g. for developing models of disease risk^1,2^, disease progression^3-6^ or different disease stages^7^.

Each of these studies has unavoidably certain biases due to inclusion/exclusion criteria or over-representation of specific geographic regions and ethnicities. Moreover, usually neither the same clinical outcome measures nor the same molecular data are systematically collected in different studies of the same disease. Accordingly, compilation of a comprehensive view of a specific disease requires to analyze and compare multiple studies. A large number of clinical studies is nowadays conducted in the context of drug development in pharmaceutical research. However, legal and ethical constraints typically do not allow for sharing sensitive patient data outside the organization that is responsible for the study. Even within one and the same organization the same reasons sometimes prevent data sharing. In consequence there exist data “silos”, which slow down the overall scientific progress in translational research.

In this paper, we suggest the idea of a virtual cohort (VC) to address this limitation. Our idea is to describe a longitudinal patient cohort with the help of a statistical model (namely a Bayesian Network) in conjunction with deep learning techniques (sparse autoencoders). This allows for simulating subjects that are sufficiently similar to real ones and can be shared with other organizations. Researchers could then develop models and generate hypotheses based on VCs that can later on be tested with the help of real data within their own organization. Hence, we overcome the aforementioned data privacy issues regarding sharing of real patient data. Moreover, we demonstrate that VCs open the opportunity to simulate scenarios, which have not been observed in reality (e.g. a certain shift towards a more healthy population). Our work should at this point be discriminated from existing work on virtual trial simulation, which mostly focuses on pharmacokinetic-pharmacodynamic (PKPD) modeling in clinical study design and typically involves mechanistic modeling of well understood biological processes^8^. In contrast, the idea behind our VC concept is to cover the complex interplay of different biological scales and data modalities (clinical, genomic, imaging, etc.) within one modeling framework. This allows for generating virtual patient trajectories that are highly similar to real ones with respect to relevant characteristics. We consider Bayesian Networks (BNs) as interesting candidates for realizing this ambition, because they allow for integrating highly heterogeneous data within one modeling framework^9^ while allowing to address one of the main challenging aspects of clinical study data, namely high numbers of missing values. Moreover, BNs belong to the family of generative models and hence can be used to simulate virtual patient trajectories after model fitting^10^.

From a methodological point of view one of the main challenging issues that we address in this paper is that many patients typically drop out of a study at some point, e.g. due to symptom worsening. Most authors thus focused their modeling efforts only on patients that were observed till the end of a study^3^, which can result into drastic loss of data and potential model biases. One of the important aspects of our proposed approach is therefore a mechanism, which directly incorporates systematic missingness of data in a longitudinal patient study into a BN model. A further distinction point of our work is the combination with deep learning techniques, specifically autoencoder networks, to reduce the dimensionality of our data. It further enables the application of BN structure learning to data of realistic sample size at reasonable computational cost. Our approach can be interpreted as a special type of Module Network^11^, which allows for incorporating arbitrary multi-scale, multi-modal data without making specific distribution assumptions. We suggest a conservative approach to simulate virtual patients, which allows for scoring virtual subjects compared to the distribution of real patients and potentially rejecting them, if they might be considered as outliers. Using this approach we demonstrate that our simulated Alzheimer’s (AD) and Parkinson’s Disease (PD) VCs cannot be reliably discriminated from real patients in ADNI and PPMI. Furthermore, our method can be used to simulate a VC for a situation that has not been observed in the real data, e.g. a less cognitively impaired AD cohort.

## Results

### Used datasets

#### ADNI

Data were obtained from the Alzheimer’s Disease Neuroimaging Initiative (ADNI) database (adni.loni.usc.edu). The ADNI was launched in 2003 as a public-private partnership, led by Principal Investigator Michael W. Weiner, MD. The primary goal of ADNI has been to test whether serial magnetic resonance imaging (MRI), positron emission tomography (PET), other biological markers, and clinical and neuropsychological assessment can be combined to measure the progression of mild cognitive impairment (MCI) and early Alzheimer’s disease (AD). For up-to-date information, see www.adni-info.org.

The study includes 417 cognitively normal patients, 106 subjects with significant memory concern, 310 subjects with early mild cognitive impairment, 562 subjects with late mild cognitive impairment and further 342 subjects, which were diagnosed with AD at the beginning of the study. In this work, we used longitudinal data from 689 patients that were initially either diagnosed as AD (*n *= 342) or converted into AD patients during the study. ADNI data includes single-nucleotide polymorphism (SNP) based genotype, APOE4 status, CSF (cerebrospinal fluid) biomarkers, volume measurements of seven brain regions as well as different clinical and neuropsychological test results. In addition to the 7 brain volume measurements provided in the original ADNIMERGE dataset, we calculated 68 cortical brain region volumes from raw images using Desikan parcellation (details in “Methods” section). Out of more than 300,000 SNPs that have been commonly measured in the ADNI1 and ADNI2/GO phases of the ADNI study we focused on 110, which have previously been implicated as relevant in the transition of a normal/cognitively impaired state to AD^2^. We grouped all features measured in ADNI into brain volumes, cortical brain regions, cognition tests, CSF markers, genotype (SNPs + APOE4 status), demographic features and baseline diagnosis (see exact definitions in Supplements). We generally discarded features with more than 50% missing values, which reduced the number of visits modeled by our approach to baseline, month 6, month 12 and month 24.

#### PPMI

Data were obtained from the Parkinson’s Progression Markers Initiative (PPMI) database (www.ppmi-info.org/data). For up-to-date information on the study, visit www.ppmi-info.org. PPMI consists of multiple cohorts from a network of clinical sites with the aim to identify and verify progression markers in PD. It is a multi-modal, longitudinal observation study with data collected using standardized protocols^12^. PPMI comprises of eight cohorts with different clinical and genetic characteristics.

Here we used data of 362 de novo PD patient cohort. These untreated subjects were diagnosed with PD for 2 years or less and showed signs of resting tremor, bradykinesia, and rigidity during the last 2 years. The dataset contains 831 clinical variables, which we categorized into 12 groups, such as patient demographics, patient PD history, imaging, non-motor symptoms, CSF markers and UPDRS (see complete list and exact definition in Supplements). PPMI assesses clinical variables at baseline and 11 follow-up visits. Noteworthy, some variables were assessed irregularly and not for all patients, yielding missing values. We generally discarded features with more than 50% missing values for modeling purposes. Accordingly, there were 12 time points included into our model, but not all variables were available at each time point (see Supplements).

### Motivation and overview about developed method

In a naive way one might consider simulating a VC by assuming all variables in a clinical cohort to follow a Gaussian distribution and inferring population parameters from reported summary statistics (means, standard deviations) as parameters. However, the Gaussian distribution assumption does not hold true in many cases, e.g. for categorical variables. A further big disadvantage is the ignorance of statistical dependencies between variables. For example, sampling age and height of persons independently could result into a virtual subject that is 6 months old and has a height of 1.80 m.

BNs explicitly describe conditional statistical dependencies between variables and thus address this issue. Moreover, they are generative models, i.e. represent a statistical distribution, from which data can then be drawn. However, in reality the true BN structure is only partially known. Learning the network structure from data is in principle possible, but computationally NP hard^28^. The number of possible network structures between *n* variables grows super-exponentially with *n*, which raises severe concerns regarding the identifiability of the true edges from limited number of patients in clinical studies. Moreover, the limited number of patients can yield overfitting of parameters of the BN.

**Figure 1:**
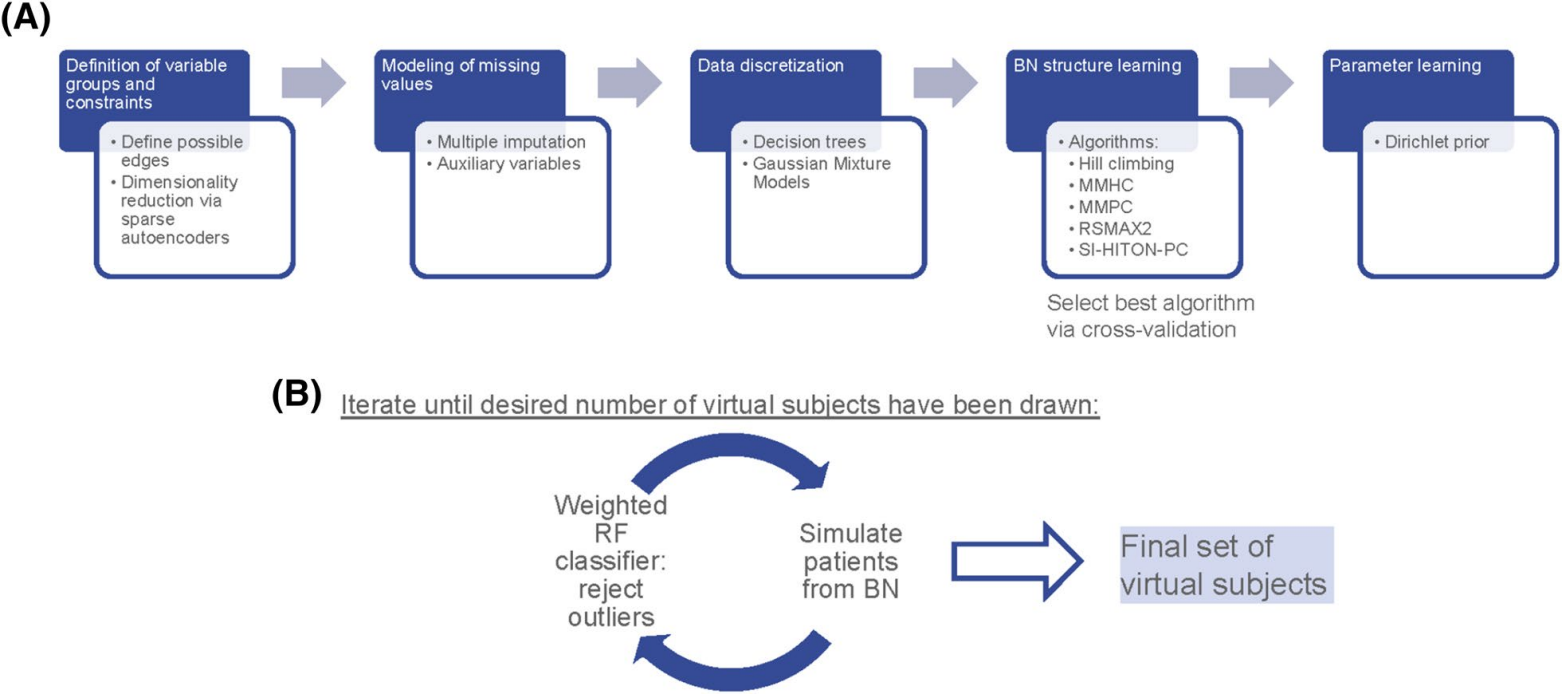
Overview about our modeling approach for longitudinal patient cohorts: (**A**) Approach to estimate BN, including dimensionality reduction via sparse autoencoders and modeling of missing data. (**B**) Conservative approach to simulate virtual patients.

To address these concerns we here developed a combined deep learning and BN based approach to simulate longitudinal multi-modal, multi-scale VCs. Our approach, summarized in Fig. 1, starts with the definition of variable groups and potentially allowed dependencies between these groups (Figures S1, S2). This step significantly reduces the number of parameters and possible BN structures. Moreover, we model missing values (specifically including those that cannot be regarded as missing by randomness). To aggregate data on the level of variable groups we use sparse autoencoders, which do not make any assumptions about the underlying statistical distribution. Next we discretize the data to enable efficient BN structure and parameter learning for arbitrary statistical distributions and non-linear dependencies between variable groups. The fully parametrized BN is then used to draw virtual subjects. Further details about our approach, including the relationship to existing BN methods, are described in the “Methods” section of this paper.

A question that is critical for the utility of a VC is, in how far simulated data statistically resembles real subjects. The definition of a quality criterion for VCs is difficult, and there is probably not a unique answer. In this work we propose a conservative approach, in which we iteratively train a weighted Random Forest (RF) classifier^13^ that allows us to score and potentially also to reject virtual subjects. Our method is described in detail in the “Methods” section of this paper.

### Virtual AD and PD patients look realistic

To validate our VC generation scheme we generated the same number of virtual as well as real patients for ADNI and PPMI and then asked whether a conventional RF classifier was able to separate between virtual and real subjects within a 10 times repeated 10-fold cross-validation scheme. That means we sequentially left out 1/10 of subjects and trained a RF on the remaining subjects to learn the discrimination between real and virtual subjects. We used the left out portion of the data to assess the prediction performance of the RF. We used the partial area under ROC curve (pAUC) at a pre-specified true positive rate of 90% for real patients as a measure of the prediction performance. That means we looked at the area under the ROC curve at which the detection rate for real patient was between 90% and 100%. This was done to account for the fact that misclassification of a virtual patient as real would be far less relevant as the other way around. Following the implementation in R-package pROC^14^ the pAUC is a measure in the interval [0, 1], where 0.5 represents chance level.

We compared our proposed conservative simulation approach to drawing virtual patients directly from the BN model fitted to the entire data. Figures S7 and S8 indicate a clear benefit of our proposed method as the pAUC value is lower for the conservative approach compared to directly drawing virtual subjects from the Bayesian Network.

**Figure 2:**
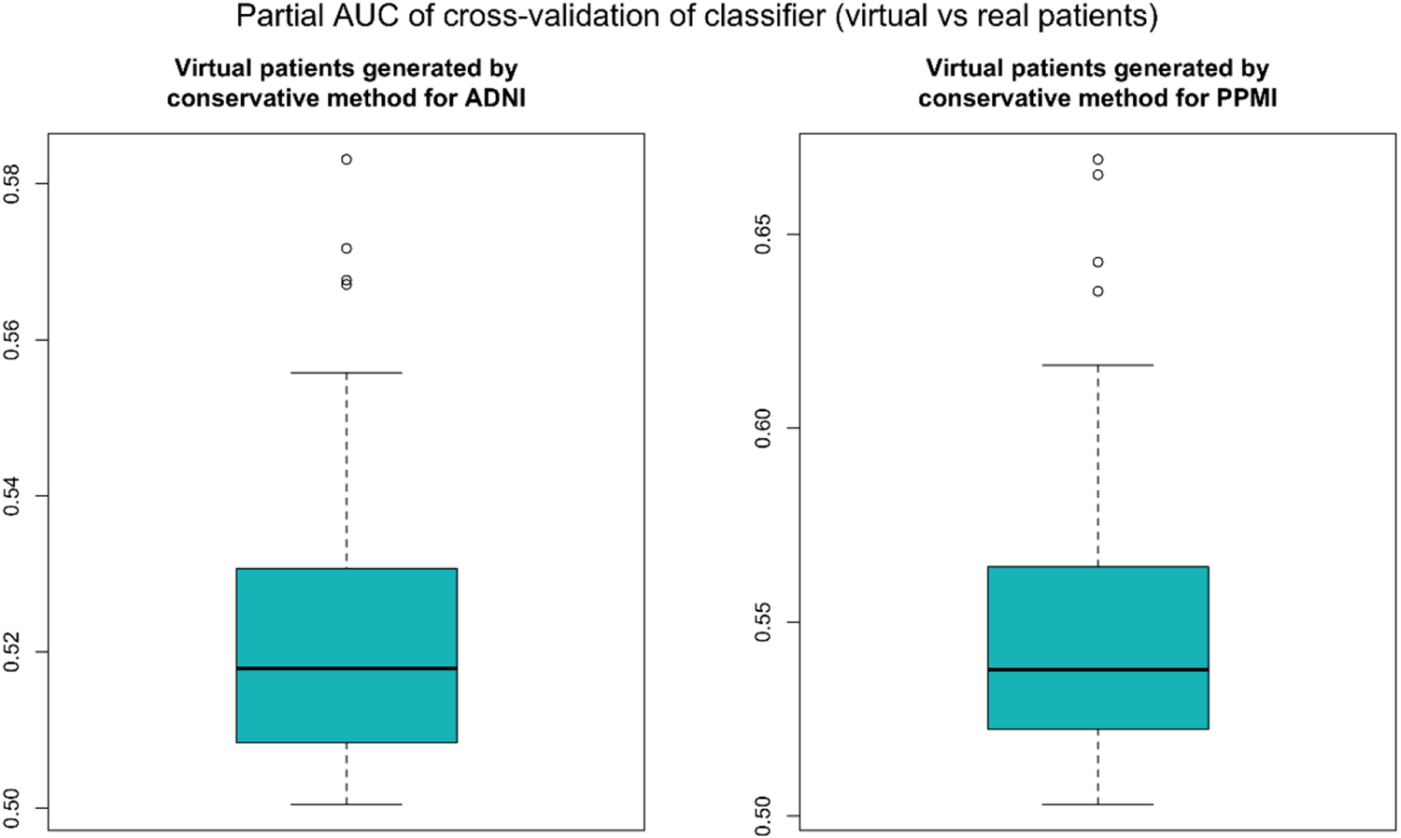
Performance of a random forest to correctly identify virtual subjects, measured via the partial area under ROC curve (pAUC) at a pre-specified detection rate of $\ge$90% for real patients. The pAUC was assessed on test sets within 10 repeats of a tenfold cross-validation procedure. Accordingly, boxplots show the distribution of the tenfold cross-validated pAUC that was obtained from 10 repeats of the cross-validation procedure.

**Figure 3:**
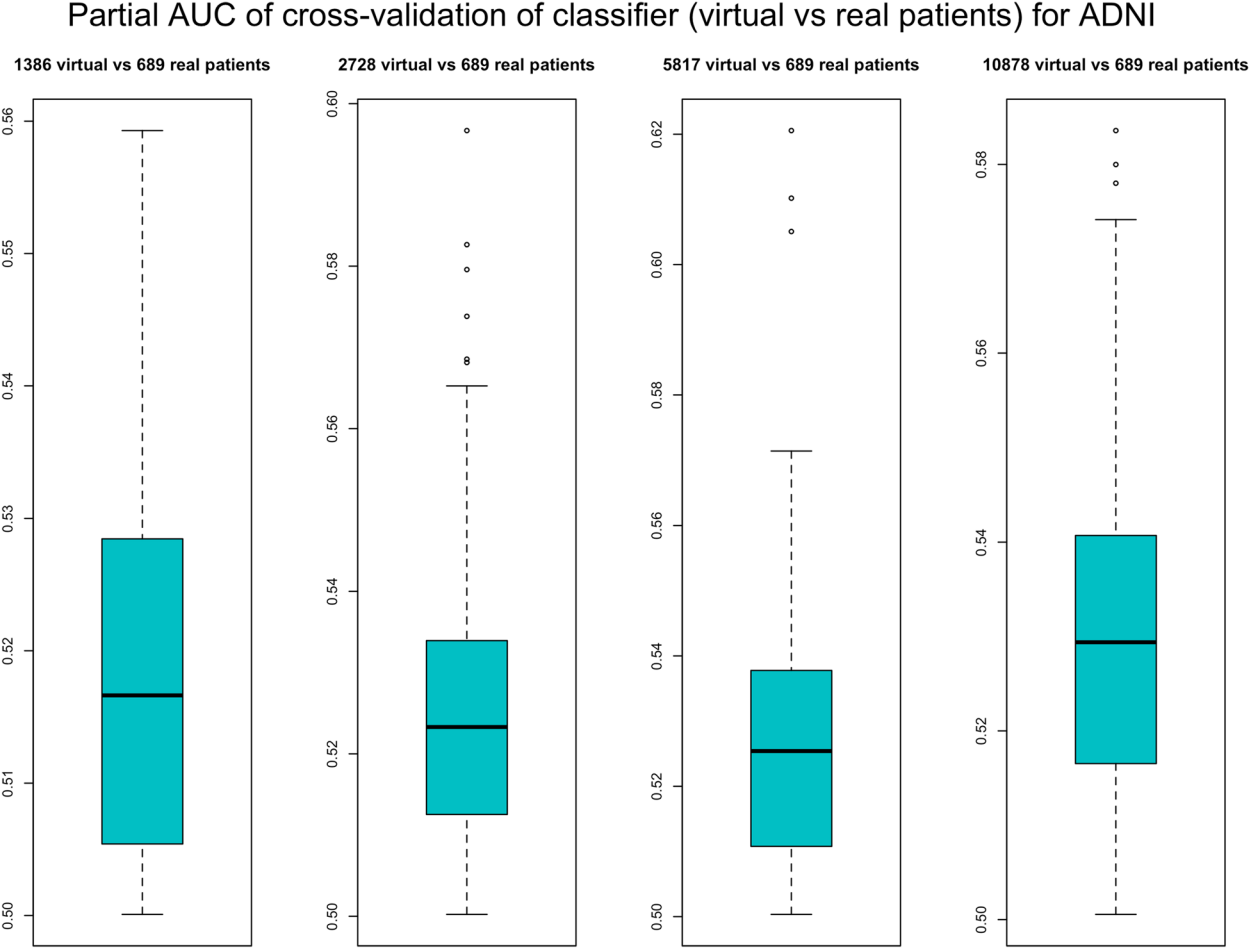
Performance of random forest classifier to correctly identify a given number of real ADNI patients among virtual subjects. The performance was measured via the partial area under ROC scurve (pAUC) at a pre-specified detection rate of $\pm$90% for real patients. The pAUC was assessed on test sets within 10 repeats of a tenfold cross-validation procedure. Accordingly, boxplots show the distribution of the tenfold cross-validated pAUC that was obtained from 10 repeats of the cross-validation procedure.

**Figure 4:**
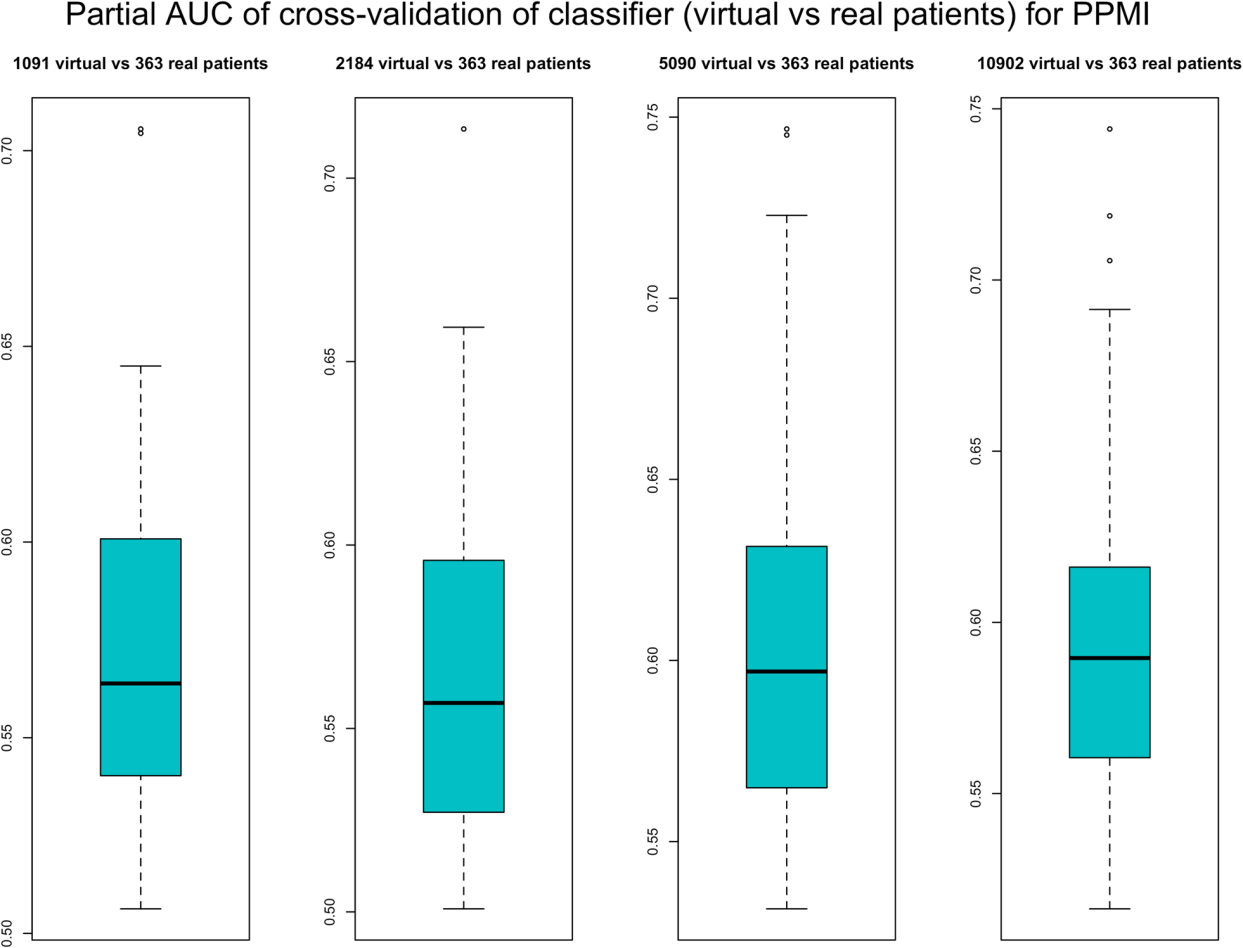
Performance of Random Forest Classifier To correctly identify a given number of real PPMI patients among virtual subjects. The performance was via the partial area under ROCcurve (pAUC) at a pre-specified detection rate of $\ge$90% for real patients. The pAUC was assessed on test sets within 10 repeats of a tenfold cross-validation procedure. Accordingly, boxplots show the distribution of the tenfold cross-validated pAUC that was obtained from 10 repeats of the cross-validation procedure.

Figure 2 demonstrates that for both, ADNI and PPMI, the cross-validated classification performance to detect virtual patients is not clearly better than chance level. This situation did also not change significantly when generating a varying number of more virtual than real patients (Figs. 3, 4).

**Figure 5:**
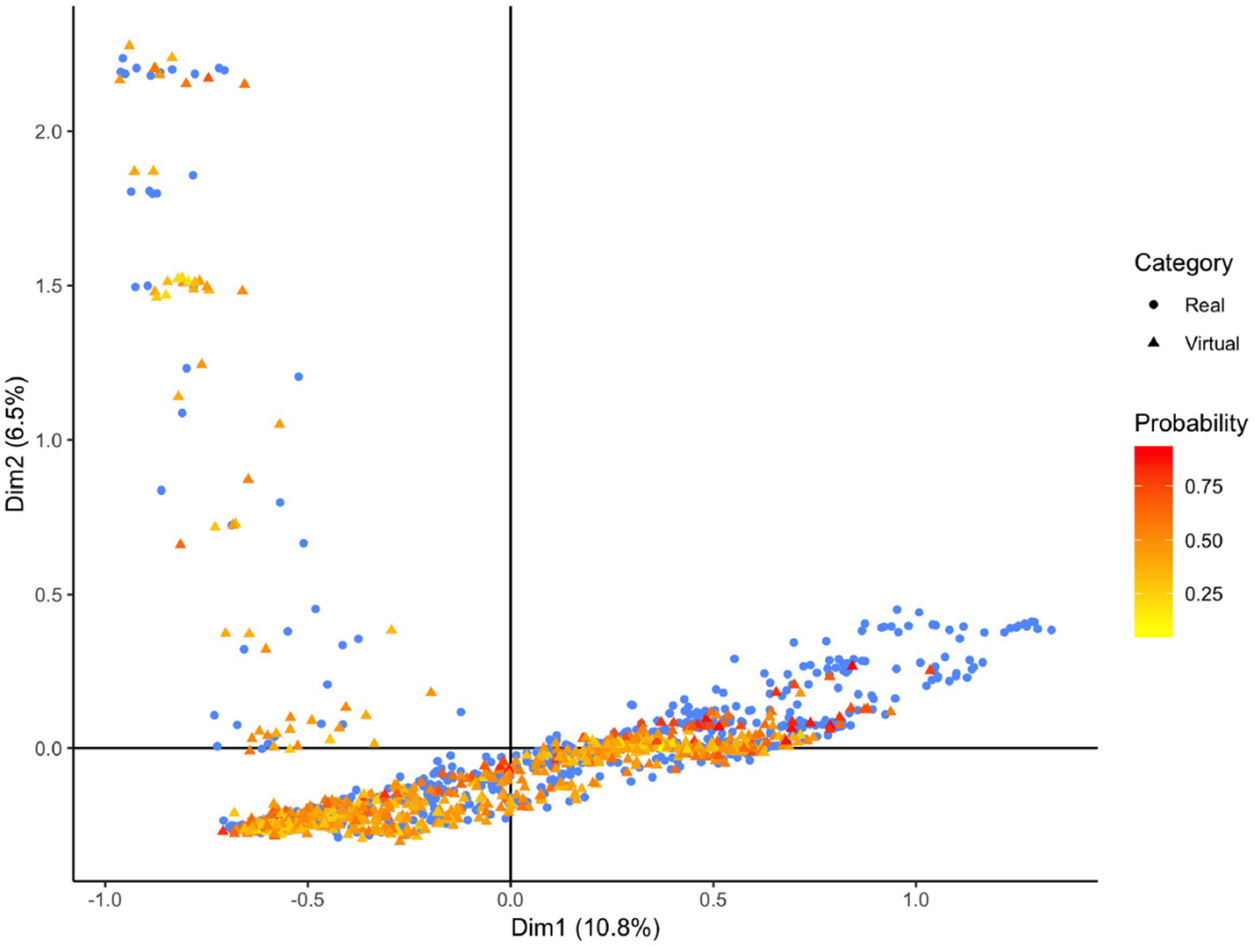
Multiple correspondence analysis plot of real ADNI and virtual patients. The probability of a virtual patient to belong to the real data is shown via a color code.

**Figure 6:**
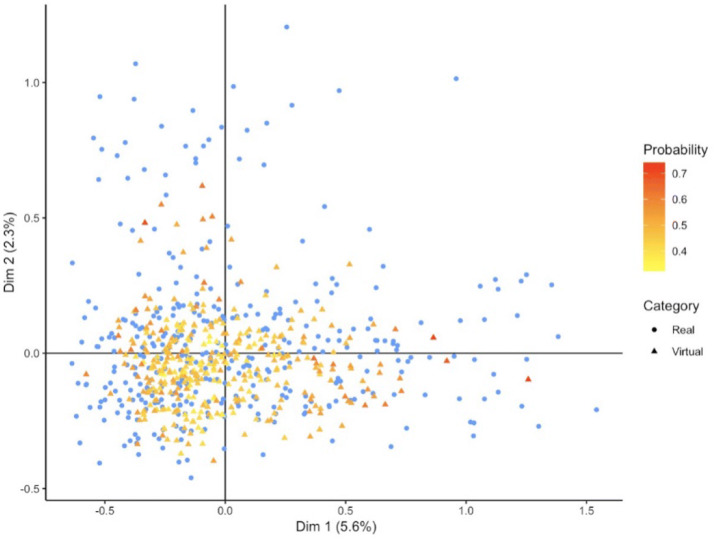
Multiple correspondence analysis plot of real PPMI and virtual patients. The probability of a virtual patient to belong to the real data is shown via a color code.

In addition to the RF based multivariate evaluation we inspected, marginal variable distributions (Figures S9, S10). We assessed separately for each variable via a *χ*^2^-test whether the null hypothesis of virtual and real patient samples coming from the same statistical distribution could be rejected (homogeneity test). Results shown in Tables S3, S5 demonstrate that after multiple testing correction univariate differences between virtual and real patient samples were in the vast majority of cases insignificant, which is in agreement to the findings from our RF based evaluation. Note that statistical significance in general is dependent on sample size and always has to be interpreted together with effect size. Thus, we think that our suggested RF based evaluation constitutes a more direct and interpretable way to assess the quality of a VC. At this point we should mention that our suggested RF method assigns to each virtual patient a confidence score, namely the probability to belong to the class of real patients. Figures 5 and 6 visualize these confidence scores with a color code in a multiple correspondence analysis plot (a technique similar to PCA that is devoted to discrete data^15^). Altogether, we conclude that our proposed approach results into VC simulations that exhibit a sufficient degree of similarity to real patients.

Our suggested approach relies on an initial data discretization step followed by BN learning and VC simulation. We compared the VC generated by this approach to an alternative one, in which the data discretization was omitted and instead a hybrid continuous/discrete BN was directly learned from the data (details in “Methods” section). As demonstrated by Figure S11 this approach was clearly inferior to our suggested one, because it resulted in a significantly better discrimination of virtual from real patients. This was likely due to the false assumption made by the hybrid BN that all continuous features would follow a Gaussian distribution. Hence, hybrid BNs were not considered further during the rest of this paper, and all presented results refer to our suggested method.

### Bayesian network structures reflect expected causal associations

**Figure 7:**
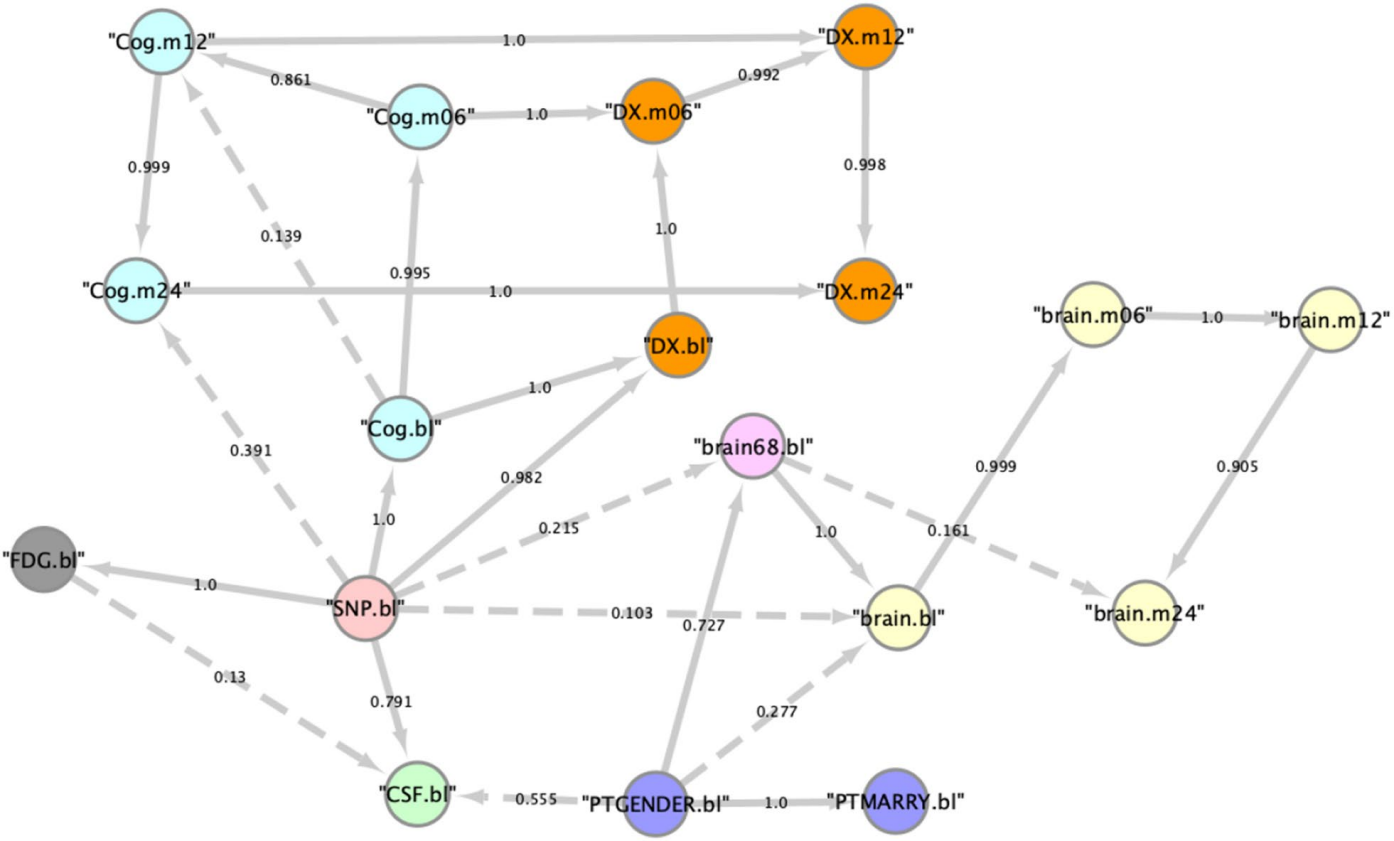
Variable dependencies identified in ADNI dataset in more than 100/1,000 bootstrapped Bayesian network reconstructions (dashed lines; relative frequency = edge label). Solid edges indicate variable dependencies that are found commonly in bootstrapped Bayesian Network reconstruction and the final Bayesian Network topology.

To gain a better understanding of the variable dependencies learned by our BN models we performed a non-parametric bootstrap^16^^.^ That means we sampled for 1,000 times n patients (with n being the number of patients in each dataset) with replacement, and for each of these 1,000 bootstrap samples we learned a complete Bayesian Network structure. We then counted the relative frequency of observing a particular edge. Figure 7 depicts the network structure for ADNI and Figure S6 depict the structure learned by PPMI data. The description of individual variables is illustrated in Table S3 and Table S5.

As expected, edges connecting variables, which represent the same group of features (e.g. UPDRS, CSF biomarkers, brain volume measurements) at different visits were inferred more stable than edges between different variable groups. That means BN structure learning was able to stable learn longitudinal dependencies in the data. This is e.g. marked by the connections between variables DX.BL (baseline diagnosis), DX.6 (diagnosis at 6 months), DX.12 (diagnosis at 12 months) and DX.24 (diagnosis at 24 months) in ADNI. Clinical diagnoses at each time point is dependent on cognitive impairment scores at the same time point, because the clinical diagnosis of dementia in practice is done on the basis of such tests.

In addition, in ADNI stable connections between genotype (SNPs) and baseline diagnosis, cognitive impairment scores (Cog.bl) and amyloid PET scan diagnostics (FDG) were found. We investigated the relative influence of individual SNPs in the sparse autoencoder network output to understand these connections better. This was done via the random input permutation method described in Gedeon et al.^17^, see “Methods” section for more details. Altogether there was a non-zero influence of all 110 SNPs plus APOE4 status in the SNP group (see Supplementary Excel file). The most relevant SNP (rs9384488) has been associated with quantitative global cortical amyloid-$\upbeta$ load^18^. Amyloid-$\upbeta$ plaques are one of the hallmarks of Alzheimer’s Disease, and amyloid-$\upbeta$ measurements are part of the CSF variable group, hence providing an interpretation of the SNP → CSF edge in our BN as well as SNP → FDG.

In PPMI the edge of UPDRS1 to non-motor symptoms reflects (found in about 500/1000 BN reconstructions) the fact that the UPDRS scoring system comprises three parts, and the first part captures non-motor symptoms (cognitive function, behavior and mood)^19^. Similarly, the stable edge between non-motor symptoms and RBD (REM Sleep Behavior disorder score) can be explained by the fact that sleeping disorder assessment is part of non-motor symptom related variables in PPMI.

In summary, BN structures learned by our models reflected expected variable dependencies in both datasets.

### Simulating an intervention in a VC

As pointed out before the final BN structure learned from ADNI represents expected dependencies between variable groups and indeed all of these dependencies can be regarded as causal (Fig. 7, solid edges): In particular note that the effect of genotype (SNPs) on cognitive assessment scores (Cog.bl), amyloid PET scan diagnostics (FDG.bl) and baseline diagnosis can only be interpreted causally. Likewise, the influence of gender on subcortical brain volumes can only be interpreted causally, although there potentially exist mediators such as longevity (women on average live longer than men).

To further exemplify the use of our causal BN, we simulated an intervention for dementia and Mild Cognitively Impaired (MCI) patients at baseline that shifted their cognition scores (ADAS11, ADAS13, MMSE, CDRSB, FAQ, RAVLT) towards the median score of the cognitively normal patients (*n *= 423), e.g. via a drug. Dementia is the most severe stage in AD patients and MCI is the stage between cognitively normal decline and dementia decline. We encoded perturbed cognition scores via the autoencoder model that we had trained earlier on cognition scores of real patients. We then simulated the same number of virtual subject trajectories as real patients while conditioning on the shift in study baseline cognitive assessment scores. That means we used the perturbed cognition scores of real patients and then sequentially drew data for each dependent variable using the conditional probability tables (i.e. BN parameters) learned by our model. This implies that the intervened node becomes statistically independent from its parents, i.e. all incoming edges of variable Cog.bl are deleted in the intervened network^20^.

**Figure 8:**
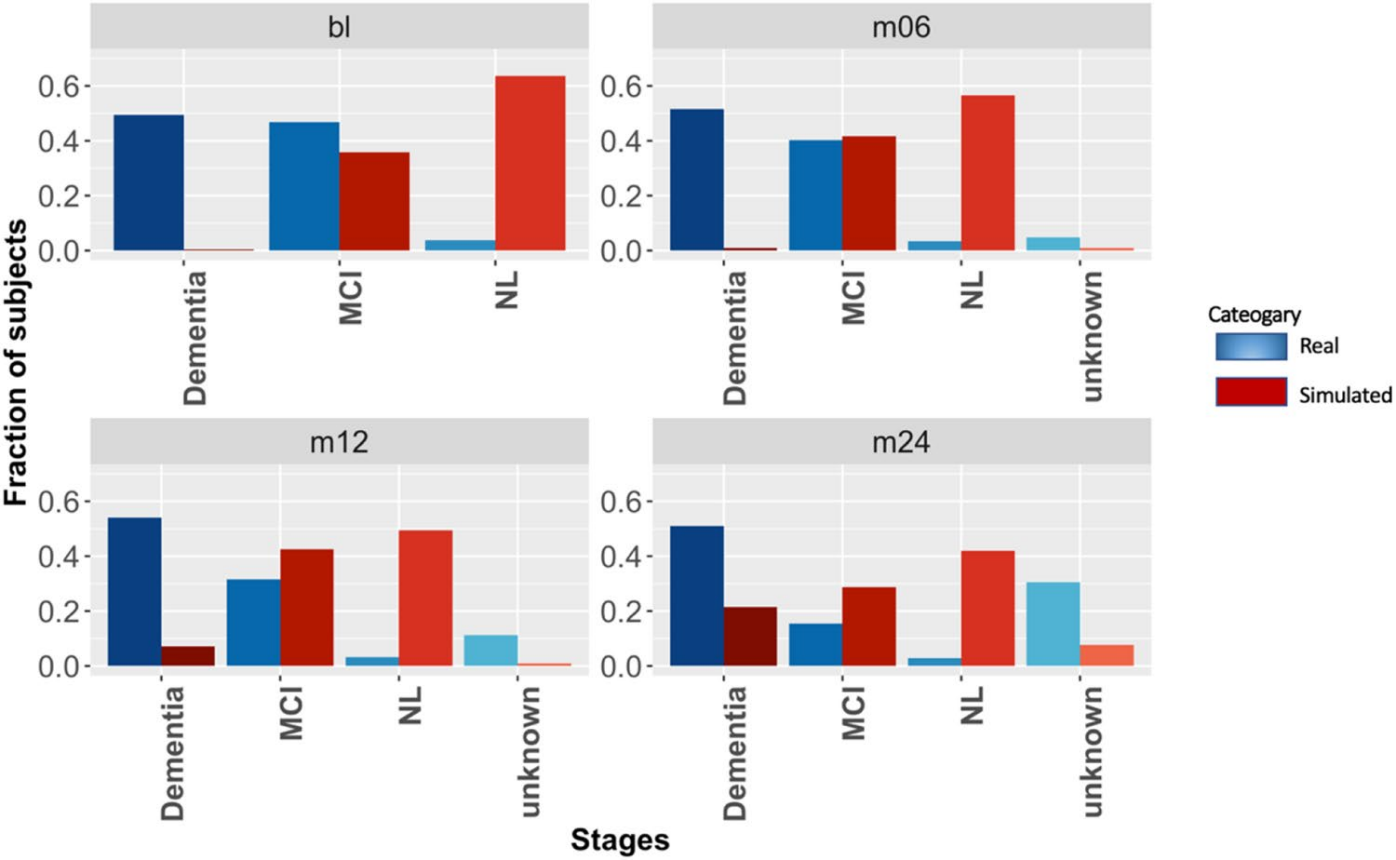
Simulation of a VC with an intervention: The figure shows diagnostic labels of 689 real ADNI patients (red) and of a simulated cohort of the same size (blue) at different visits. The cognitive assessment scores of the simulated cohort have been shifted at baseline. MCI, mild cognitive impairment; NL, cognitively normal; unknown, unknown diagnosis/diagnosis not reported.

Figure 8 demonstrates, how the effect of our counter-factual improvement of cognition scores at baseline resulted into an expected significant shift of diagnoses towards “cognitively normal” or “mild cognitively impaired” throughout the study. Hence, our simulation of a “perturbed” ADNI cohort underlines the validity of our BN model.

Altogether this example underlines the validity of our BN models and demonstrate the possibility to—at least qualitatively—study intervention effects in silico.

## Discussion

To our knowledge this paper demonstrates for the first time a realistic simulation of virtual clinical subject trajectories across multiple biological scales and data modalities outside the area of mechanistically well understood biological processes. This was achieved via a combination of deep learning techniques (sparse autoencoders) to significantly reduce the input dimensionality of our data and BN learning. The BN allows us to model missing data (specifically MNAR) and serves as a generative model, from which patient trajectories can be drawn. We proposed a conservative approach to simulate VCs, which allows to score and reject virtual subjects, if they appear different from real ones. Using this method we showed that virtual and real patient trajectories are highly similar, but not identical. Hence, our proposed approach opens the possibility to build virtual and at the same time realistic versions of clinical studies across multiple disease areas in the future. These virtual studies could then be shared with the larger research community, even, if the raw data cannot because of legal or ethical constraints. Hence, our method could help to unlock one of the key bottlenecks in biomedical research in data scarce disease areas.

We also showed that our VC approach allows for simulating interventions and studying their downstream effects in a qualitative manner in silico. Such an approach could help the design of future clinical studies, because it allows to assess, which variables or variables groups are more likely to show differences after a planned intervention (e.g. with a drug).

Our proposed approach is not without limitations: Modular BN structure learning requires to define variable groups and constraints on the network structure, which implies a detailed understanding of the data. Moreover, we typically need to discretize input data to account for non-linearities between input variables while making BN structure and parameter learning at the same time computationally efficient. BN structure and parameter learning requires sufficiently large datasets that are representative for the disease population. In addition, our method uses a sparse autoencoder based aggregation of input features into variable groups, which naturally implies computational costs and a certain loss of information. Drawings of virtual patients from the modular BN model thus make the re-identification of real patients from the training data relatively unlikely. However, in its current implementation our approach does not provide strict theoretical guarantees for this situation. But, we like to point out that privacy preserving training of neural network models is possible in principle^21^. Training all sparse autoencoders via the modified stochastic gradient descent algorithm proposed by Abadi et al. provides theoretical guarantees on data privacy of our entire modular BN model. In future work we will explore this aspect further and make an according implementation available. Altogether, the work presented here can only be seen as a first proof of concept for the idea of simulating realistic multi-scale, multi-modal VCs, and further methodological advancements are necessary.

## Methods

### Bayesian networks for non-stationary mixed static and longitudinal data

The key ingredient of our proposed approach is a Bayesian Network (BN) describing longitudinal patient trajectories in a multi-modal, multi-scale manner: Let *G *= (*V*,* E*) be a directed acyclic graph (DAG) and *X *= (*Xv*)*v *∊ *V* a set of random variables indexed by nodes in *V*. *X* is called a Bayesian network with respect to *G*, if the joint distribution *p*(*X*_1_*, X*_2_, ...,* X*_*n*_) factorizes according to:

$$p(X_{1} = x_{1} , \, X_{2} = x_{2} , \, \ldots , \, X_{n} = x_{n} ) \, = \prod\limits_{v \in V} {p(X_{v} = x_{v} | \, X_{pa(v)} = x_{pa(v)} )}$$

where *pa*(*v*) denotes the parents of node *v* and *xpa*(*v*) their joint configuration^10^. For a given node *v* we summarize the set of associated conditional probabilities into a parameter vector $\theta$*v*, and these parameter vectors are assumed to be statistically independent for different nodes *v*, *v*′.

In our situation there exists a subset $\tilde{X} \subset X$ such that measurements are time dependent, i.e. ${\tilde{\mathbf{x}}} = (\tilde{x}({1})), \ldots ,\tilde{x}(T))$ with *T* being the number of visits. Dynamic Bayesian Networks^22^ usually deal with thiss situation by implicitly unfolding the BN structure over time, i.e. introducing for each visit *t* a separate copy $\tilde{X}$(*t*) of $\tilde{X}$ while requiring that edges always point from time slice *t* to time slice *t* + 1 (corresponding to a first order Markov process). This implicit unfolding assumes a stationary Markov process, i.e. parameters $\theta$ do not change with time. In our setting this assumption is most likely wrong, because patients change in their disease outcome during the course of a study, i.e. $p(\tilde{X}\left( t \right)|\tilde{X}(t \, - {1})) \ne p(\tilde{X}(t + {1})|\tilde{X} \left( t \right))$. Hence, we here use an unfolding strategy, in which we explicitly use different copies $\tilde{X}$ (*t*) for each time point.

### Dealing with missing data

One of the key challenges with longitudinal patient data is missing values, which can result due to different reasons: (a) patients drop out of a study, e.g. due to worsening of symptoms; (b) a certain diagnostic test is not taken at a particular visit (e.g. due to lack of patient agreement), potentially resulting into missing information for entire variable groups; (c) unclear further reasons, time constraints, data quality issues, etc. From a statistical point of view these reasons manifest into different mechanisms of missing data^23,24^:

1. *missing completely at random (MCAR)* The probability of missing information is not related to either the specific value which is supposed to be obtained or other observed data. Hence, entire patient records could be skipped without introducing any bias. However, this type of missing data mechanism is probably rare in clinical studies.
2. *missing at random (MAR)* The probability of missing information depends on other observed data, but is not related to the specific missing value which is expected to be obtained. An example would be patient drop out due to the worsening of certain symptoms, which are at the same time recorded during the study.
3. *missing not at random (MNAR)* any reason for missing data, which is neither MCAR or MAR. MNAR is problematic, because the only way to obtain unbiased estimates is to model missing data.

Missing values in ADNI and PPMI data are most likely a combination of MAR and MNAR mechanisms. In general, multiple imputation methods have been proposed to deal with missing data in longitudinal patient data^24^. Specifically for MNAR it has been suggested to explicitly encode systematic missingness of variables or variable groups via dedicated indicator variables^25^. In our BN framework these auxiliary variables are fixed parents of all nodes, which contain missing values in a “systematic” way, and there are no further edges learned for auxiliary variables. We introduced one auxiliary variable for each variable group and visit to account for patient drop-out, i.e. MNAR. Moreover, in case of features that are assessed at different visits we enforced auxiliary variables to point from one to the next visit. For example, in ADNI dataset we introduced auxiliary variables for brain volume measurements at baseline, visit 6 months, visit 12 month, visit 24 month. Accordingly, the auxiliary variable for the feature “brain.bl” was also a parent of the auxiliary variable for the feature “brain.m06” (Figure S1). Details about the precise definition of auxiliary variables used in our work can be found in the Supplementary material.

A consequence of auxiliary variables is that they make parameter estimates conditionally dependent on the missingness information, hence accounting for potential biases of the multiple imputation (due to hidden confounding factors), which in our case was conducted via the missForest method^26^. Briefly, missForest is a non-parametric multiple imputation approach for mixed data types, which uses Random Forests to iteratively predict missing values in each variable based on information in all other features. Starting from initial guesses of missing values, the method runs through several iterations of predictions until convergence of missing values can be observed. Stekhoven and Bühlmann demonstrated that specifically for mixed discrete/continuous data missForest compares favorably against a number of alternative imputation technique, which was the reason for choosing this particular method here.

### Imposing constraints on the network structure

Most edges in the BN structure are not known and hence need to be deduced from data. An important question is, in how far the learned structure then reflects existing causal relationships. Indeed, if the BN is faithful to the underlying statistical distribution (i.e. models it correctly), then the true causal network is known to be part of a class of equivalent graph structures, called *class partially directed acyclic graph* (CPDAG)^10,27^. Under the above mentioned assumptions, the CPDAG has the same skeleton as the true causal graph, but may leave some edges undirected. Hence, in practical applications, it is important to restrict the CPDAG equivalence class as much as possible by prior knowledge to allow correct orientation of as many edges as possible. In our case we specifically imposed the following constraints for BN structures:

- Demographic and other clinical baseline features (age, gender, ethnicity) can only influence other features, but they are not themselves influenced.
- Medical history can depend on motor, non-motor and other clinical features.
- Imaging features can be related to each other, but they don’t influence other features.
- Clinical diagnosis in AD is dependent on cognitive assessment scores, but not vice versa
- Clinical outcome measures (e.g. UPDRS for PD) can influence imaging and—in Parkinson—they can be mutually correlated with assessment of non-motor symptoms.
- Biomarkers, including genomic features, can influence all features, except for clinical baseline features.
- Longitudinal features must follow the right temporal order, i.e. there are no edges pointing backwards in time.
- An auxiliary variable which is created on the basis of missingness of certain feature can only influence it’s corresponding feature and the auxiliary variable for the same feature at the next time point (see last section and Figure S1).

Figures S2, S3 schematically depict the set of potentially allowed edges, which we defined between variable groups for the PD and AD datasets used in this work.

### Dimensionality reduction via modular BNs using sparse autoencoders

Learning the true CPDAG structure is NP hard^28^, which requires to limit the space of potential networks as much as possible. This is of particular importance in a situation with many variables and a limited sample size, such as ours. Module Networks have been introduced as a way to address such a situation^29^. The key idea in Module Networks is to group variables into modules, which share parameters. During the BN structure learning process, only edges between modules are learned. In our case, variable comprised e.g. imaging related features, plasma biomarkers, SNPs, medical history, cognition scores, etc. The exact definition of variable groups differs between datasets used and is shown in the Supplementary material (Tables S3, S5). The key question is, how to learn and encode a shared distribution for a module. In their original publication Segal et al. relied on the assumption of normally distributed data (such as gene expression) and employed decision trees to represent modules^29^^.^ In this work we used sparse autoencoders, which can weigh the influence of different variables on to the aggregate module score. Furthermore, autoencoders do not make any distribution assumption. Briefly, autoencoders are a form of neural network, which perform non-linear dimensionality reduction^30^. An autoencoder takes a feature vector $x \in \Re^{d}$ as input and transforms/encodes it to a hidden representation $\tilde{x} \in \Re^{q}$ via

1 $$\tilde{x} = s(Wx + b)$$

where *s*(·) is a non-linear activation function, e.g. sigmoid, rectified linear unit. Matrix *W* consists of weights and *b* is a bias vector. Several encoding steps can be performed sequentially, resulting into a deep autoencoder. The latent representation $\tilde{x}$ can be decoded/mapped back via

2 $$z = s^{{\prime }} (W^{{\prime }} \tilde{x} \, + \, b^{{\prime }} )$$

where *W′*, *b′ *are the parameters of the decoder that are not necessarily identical to the encoder. Moreover, *s′ *(·) is a non-linear activation function, which may be different from *s*(·). Autoencoders are trained to minimize the difference between reconstructions *z* and original inputs *x*.

In our case the mean squared error (MSE) was used for that purpose. Sparsity can be enforced by introducing drop-out units into the input layer of the autoencoder network^31^. In addition, we used an *l*_2_ penalty for all weights. There was a separate sparse autoencoder trained for each variable group, and several autoencoder architectures and activation function combinations were tested via a grid search (see details in Supplements). In particular the, the grid search also involved tuning of an *l*_2_ penalty and drop-out ratios of input units. Supplementary Tables S3, S5 show the MSE obtained for each autoencoded module.

To understand the influence of individual features on each of the autoencoder networks, we applied the method by Gedeon et al.^17^, which is based on the idea that the relative contribution of the *i*th input to the *j*th output of a neuron can be estimated by:

3 $$P_{ij } = \frac{{\left| {w_{ij} } \right|}}{\sum p |w_{pj}|}$$

where the sum runs over all inputs of the neuron. P_ij_ can be regarded as the weight of the edge *i *$\to$*j* in the neural network graph. Now assume that *j* itself feeds into a further neuron *k*. Gedeon suggests to estimate the overall impact of *i* on *k* by:

4 $$P_{ik} = \sum\limits_{r} {P_{ir} P_{rk} }$$

That means we take the product of edge weights along a path connecting *i* and *k* and sum over all alternative paths. The definition can directly be extended to deeper networks.

Feature influences estimated in this way can be found for all autoencoder networks in a Supplementary Excel file.

### Data discretization

Structure learning with BNs is only computationally efficient, if all variables follow a Gaussian or multinomial distribution, because then the marginal log-likelihood, integrating out model parameters, can be computed analytically^10^. Since in our case we had highly heterogeneous data, where many features were clearly non-Gaussian, we decided to perform data discretization. In case of the ADNI study (see “ADNI” section) this was done via a supervised, decision tree based approach^32^, where baseline diagnosis of patients (cognitively normal, mild cognitively impaired or Alzheimer’s Disease) was taken as label. In case of the PPMI study (see “PPMI” section) all patients had a de novo Parkinson’s Disease diagnosis. Accordingly, we here employed an unsupervised univariate clustering via Gaussian Mixture Models for discretization purposes. Both methods result into a variable number of discrete values for each feature (Tables S3, S5).

For comparison reasons we also conducted BN structure learning *without* any discretization while assuming a Gaussian distribution for each continuous variable, see details in next section.

### BN structure and parameter learning

As a consequence of the approach described previously, any established BN structure learning algorithm could be applied. In this work we used six different algorithms implemented in the R-package *bnlearn*^33^: greedy hill climbing (50 random restarts), Max-Min Hill Climbing (MMHC)^34^, tabu search^35^, Max-Min Parent Child (MMPC)^34^, 2-phase Restricted Maximization (RSMAX2)^34^ and semi-interleaved Hiton Parent Child (SI-HITON-PC)^36^. Greedy hill climbing and tabu search are heuristic score based optimization approaches, whereas MMPC and SI-HITON-PC are constrained-based structure learning methods that try to identify the Markov Blanket of each node in the Bayesian Network. MMHC and RSMAX2 are hybrid approaches, which use ideas from both, search-and-score as well as constrained-based techniques: MMHC first learns the skeleton of the BN using the MMPC constrained-based algorithm. In a second phase edges are then oriented via a greedy hill climbing search. RSMAX2 uses for the first step the SI-HITON-PC algorithm instead of MMPC.

Selection between different BN structure learning algorithms can be done via *k*-fold cross-validation akin to conventional supervised learning^10^. That means the overall data is randomly split into *k* (here: *k *= 10) folds, and the BN structure together with its parameters successively learned from k − 1 folds. If the fitted BN correctly models the overall population (and not just the training data), the data in the left out fold should with high probability fall into the same statistical distribution that is described by the BN. This can be quantified via the negated expected log-likelihood of the test data. Accordingly, cross-validation can be used to assess the generalization ability of a BN model and to compare different structure learning algorithms on that basis.

In this work tabu search was identified as best performing BN structure learning algorithm for ADNI and hill climbing for PPMI for discretized data (Figures S4, S5). This is in agreement with recent findings that in most situations score based search methods are superior to constrained-based ones^37^ Given a learned BN topology, parameters can then be inferred using a Dirichlet prior to account for parent-child node configurations that are not observed^38^. BN structure and parameter learning was executed via the R-package bnlearn^33^.

When omitting data discretization, we end up in a BN with a mixture of Gaussian and discrete nodes (hybrid BN). To allow for a direct comparison with BN structure learning after discretization we used the same structure learning algorithm, namely tabu search. In addition, score based search algorithms have empirically found to show a more robust behavior in terms of network reconstruction accuracy than constraint based methods for mixed discrete/continuous data, specifically for smaller sample sizes^39^.

### Simulation of virtual patients

Given a BN with learned parameters, a virtual patient can be simulated by first drawing random values from parent node distributions and subsequently from their child node distributions while conditioning on the values of the parents. Each virtual patient thus corresponds to a vector of features, which follow the conditional statistical dependencies learned by the BN. If the BN is learned from discretized data, then also each virtual patient’s feature vector is discrete.

A general concern at this point is that virtual patients could show differences to real patients either due to insufficient model fit or due to the existence of confounding factors that are not part of the observed data, resulting into biases in BN parameter estimates. To account for this aspect we developed a scoring scheme, which could help to exclude unrealistic virtual patients directly after simulation. This was done by training a RF classifier^13^, which puts 100 times more weight on correctly classifying original patients than simulated ones. The weighted RF classifier assigns to each virtual subject a probability/confidence score to fall into the real patient distribution. In this way we here excluded simulated patients that showed a lower than 50% probability to fall into the real patient distribution and could thus be regarded as outlying. The whole procedure of simulating patients and excluding seemingly unrealistic ones can be run iteratively until a desired number virtual patients has been generated.

### Simulation of counterfactual interventions in Bayesian networks

Judea Pearl developed a well-established theory for modeling and simulating interventions into BNs^20^: Assume we want to predict the intervention effect of *X*_*k*_= *x* on the remaining random variabes in the BN, i.e. *P*(*X*_1_, …, *X*_*k−*1_, *X*_*k*+1_,..., *X*_*n*_* | do*(*X*_*k*_ = *x*)). Pearl demonstrated in his work that this intervention effect can be computed by estimating the conditional probability distribution *P*(*X*_1_,...,* X*_*k−*1_, *X*_*k*_+1,...,* Xn | X*_*k*_ = *x*) within a *multilated* BN, in which all incoming edges into *Xk* have been deleted.

In practice we used logic sampling^40^ for estimating the conditional probability distribution *P*(*X*_1_,...,* X*_*k−*1_, *X*_*k*+1_, ...,* X*_*n*_* | X*_*k *_= *x*) in the multilated BN.

### Calculation of cortical brain region volumes

All available MR scans (T1-weighted scans) from the ADNI database were quantified by an open-source, automated segmentation pipeline at the Erasmus University Medical Center, The Netherlands. The number of slices of the T1w scans varied from 160 to 196 and the in-plane resolution was 256 × 256 on average, yielding an overall voxel-size of 1.2 × 1.0 × 1.0 mm. From the 1715 baseline ADNI scans, the volumes of 34 bilateral cortical brain regions, 68 structures in total, were calculated using a model- and surface-based automated image segmentation procedure, incorporated in the FreeSurfer Package (v.6.0, https://surfer.nmr.mgh.harvard.edu/). Segmentation in Freesurfer was performed by rigid-body registration and nonlinear normalization of images to a probabilistic brain atlas. In the segmentation process, each voxel of the MRI volumes was labeled automatically as a corresponding brain region based on a cortex parcellation (subdivision) guide. In this case the cortical parcellation method, implemented by Desikan and Killiany in 2006^41^, was used for brain segmentation. For the subdivision of the human cerebral cortex into gyral-based regions, Desikan and Killiany manually identified the 34 cortical regions in the individual hemispheres. This information was encoded into an atlas that was utilized to automatically label ROIs. Desikan and Killiany showed that compared to manual segmentation there automated method reached an intra-class correlation coefficient (ICC) of 0.835 across all of the ROIs. The mean distance error was less than 1 mm.

## Supplementary information

Supplementary information 1

Supplementary information 2

Supplementary information 3

## References

[1] Li K, Luo S (2017). Functional joint model for longitudinal and time-to-event data: an application to Alzheimer’s Disease. Stat. Med..

[2] Khanna S (2018). Using multi-scale genetic, neuroimaging and clinical data for predicting Alzheimer’s dissease and reconstruction of relevant biological mechanisms. Sci. Rep..

[3] Hayete B (2017). A Bayesian mathematical model of motor and cognitive outcomes in Parkinson’s Disease. PLoS ONE.

[4] Qiu Y, Li L, Zhou T, Lu W (2014). Alzheimer’s disease progression model based on integrated biomarkers and clinical measures. Acta Pharmacol. Sin..

[5] Bernal-Rusiel JL, Greve DN, Reuter M, Fischl B, Sabuncu MR (2013). Statistical analysis of longitudinal neuroimage data with linear mixed effects models. Neuroimage.

[6] Conrado DJ (2018). Dopamine transporter neuroimaging as an enrichment biomarker in Early Parkinson’s Disease clinical trials: a disease progression modeling analysis. Clin. Transl. Sci..

[7] Vermunt L (2017). Duration of Alzheimer’s Disease in the preclinical, prodromal and dementia stage: a multi-state model analysis. Alzheimer’s Dement. J. Alzheimer’s Assoc..

[8] Pappalardo F, Russo G, Tshinanu FM, Viceconti M (2018). In silico clinical trials: concepts and early adoptions. Brief. Bioinform..

[9] Ahmad A, Fröhlich H (2016). Integrating heterogeneous omics data via statistical inference and learning techniques. Genom. Comput. Biol..

[10] Koller D, Friedman N (2009). Probabilistic Graphical Models: Principles and Technique.

[11] Segal E (2003). Module networks: identifying regulatory modules and their condition-specific regulators from gene expression data. Nat. Genet..

[12] Initiative PPM (2011). The Parkinson Progression Marker Initiative (PPMI). Prog. Neurobiol..

[13] Breiman L (2001). Random forests. Mach. Learn..

[14] Robin X (2011). pROC: an open-source package for R and S+ to analyze and compare ROC curves. BMC Bioinform..

[15] Greenacre M, Blasius J (2006). Multiple Correspondence Analysis and Related Methods.

[16] Friedman, N., Goldszmidt, M. & Wyner, A. Data analysis with Bayesian networks: a bootstrap approach. In *Proceedings of the Fifteenth Conference on Uncertainty in Artificial Intelligence* 196–205 (Morgan Kaufmann Publishers Inc., 1999).

[17] Gedeon TD (1997). Data mining of inputs: analysing magnitude and functional measures. Int. J. Neural Syst..

[18] Ramanan VK (2014). APOE and BCHE as modulators of cerebral amyloid deposition: a florbetapir PET genome-wide association study. Mol. Psych..

[19] Ramaker C, Marinus J, Stiggelbout AM, Van Hilten BJ (2002). Systematic evaluation of rating scales for impairment and disability in Parkinson’s Disease. Mov. Disord. Off. J. Mov. Disord. Soc..

[20] Pearl J (2000). Causality: Models, Reasoning and Inference.

[21] Abadi, M. *et al.* Deep learning with differential privacy. In *Proceedings of the 2016 ACM SIGSAC Conference on Computer and Communications Security* 308–318 (Association for Computing Machinery, 2016).

[22] Ghahramani Z, Giles CL, Gori M (1998). Learning dynamic Bayesian networks. Adaptive Processing of Sequences and Data Structures.

[23] Rubin DB (1976). Inference and missing data. Biometrika.

[24] Kang H (2013). The prevention and handling of the missing data. Korean J. Anesthesiol..

[25] Mustillo S, Kwon S (2015). Auxiliary variables in multiple imputation when data are missing not at random. J. Math. Sociol..

[26] Stekhoven DJ, Buehlmann P (2012). MissForest–non-parametric missing value imputation for mixed-type data. Bioinformatics.

[27] Spirtes P, Glymour CN, Scheines R (2000). Causation, Prediction, and Search.

[28] Chickering DM, Heckerman D, Meek C (2004). Large-sample learning of Bayesian networks is NP-Hard. J. Mach. Learn. Res..

[29] Segal, E., Pe’er, D., Regev, A., Koller, D. & Friedman, N. Learning module networks. In *Advances in Neural Information Processing Systems*, Vol. 578, 297–304 (2004).

[30] Hinton GE, Salakhutdinov RR (2006). Reducing the dimensionality of data with neural networks. Science.

[31] Srivastava N, Hinton G, Krizhevsky A, Sutskever I, Salakhutdinov R (2014). Dropout: a simple way to prevent neural networks from overfitting. J. Mach. Learn. Res..

[32] Fayyad, U. M. & Irani, K. B. Multi-interval discretization of continuous-valued attributes for classification learning. In *IJCAI* 1022–1029 (1993).

[33] Scutari M (2010). Learning Bayesian networks with the bnlearn R package. J. Stat. Softw..

[34] Tsamardinos I, Brown LE, Aliferis CF (2006). The Max-Min Hill-Climbing Bayesian network structure learning algorithm. Mach. Learn..

[35] Hong, Y., Xia, X., Le, J. & Zhou, X. Learning Bayesian network structure from large-scale datasets. In *2016 International Conference on Advanced Cloud and Big Data (CBD)* 258–264 (2016).

[36] Aliferis CF, Statnikov A, Tsamardinos I, Mani S, Koutsoukos X (2010). Local causal and markov blanket induction for causal discovery and feature selection for classification. Part I: algorithms and empirical evaluation. J. Mach. Learn. Res..

[37] Scutari M, Graafland CE, Gutiérrez JM (2019). Who learns better Bayesian network structures: accuracy and speed of structure learning algorithms. Int. J. Approx. Reason..

[38] Heckerman D, Geiger D, Chickering DM (1995). Learning Bayesian networks: the combination of knowledge and statistical data. Mach. Learn..

[39] Raghu VK, Poon A, Benos PV (2018). Evaluation of causal structure learning methods on mixed data types. Proc. Mach. Learn. Res..

[40] Henrion M, Lemmer JF, Kanal LN (1988). Propagating uncertainty in Bayesian networks by probabilistic logic sampling. Machine Intelligence and Pattern Recognition.

[41] Desikan RS (2006). An automated labeling system for subdividing the human cerebral cortex on MRI scans into gyral based regions of interest. Neuroimage.

