# Supplementary material for: Realistic simulation of virtual multi-scale, multi-modal patient trajectories using Bayesian networks and sparse auto-encoders
Source: Sci Rep. 2020 Jul 3;10:10971. doi: 10.1038/s41598-020-67398-4 (PMC7335180; doi:10.1038/s41598-020-67398-4)
Supplement: Supplementary file 3 — Supplementary information 3 [file 41598_2020_67398_MOESM3_ESM.docx]

Supplemental Material: Realistic Simulation of Virtual Multi-Scale, Multi-Modal Patient Trajectories using Bayesian Networks and Sparse Autoencoders

Meemansa Sood, Akrishta Sahay, Reagon Karki, Mohammad Asif Emon, Henri Vrooman, Martin Hofmann-Apitius, Holger Fröhlich

# Auxiliary Variables

| **Auxiliary variable** | **Target variables** |
| --- | --- |
| brainvol.bl.aux | brain.bl |
| brainvol.m06.aux | brain.m06 |
| brainvol.m12.aux | brain.m12 |
| brainvol.m24.aux | brain.m24 |
| CogScore.m06.aux | Cog.m06 |
| CogScore.m12.aux | Cog.m12 |
| CogScore.m24.aux | Cog.m24 |
| brain68.aux | brain68.bl |
| snp.aux | SNP |

Table S1: auxiliary variables defined for ADNI dataset

| **Auxiliary variable** | **Target variables** |
| --- | --- |
| CSF_aux_V00 | Abeta.42_V00, Alpha.synuclein_V00, p.Tau181P_V00, Total.tau_V00, tTau.Abeta_V00, pTau.Abeta_V00, pTau.tTau_V00 |
| Biological_aux_V00 | Biological_V00 |
| Biological_aux_V08 | Biological_V08 |
| UPDRS_aux at V01 to V11 | UPDRS1, UPDRS2,UPDRS3, UPDRS ( V01 to V11) |
| MedicalHistory_aux ( V01 to V11) | Medical History features at V01 to V11 |
| NonMotor_aux ( V02, V04, V06,V08,V10) | NonMotor features (V02, V04, V06,V08,V10) |

Table S2: auxiliary variables defined for PPMI dataset


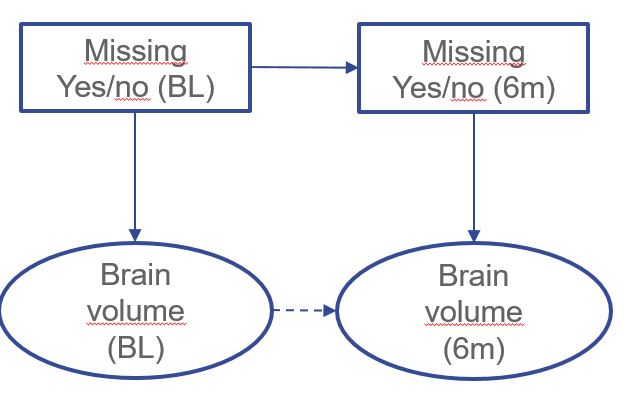


Figure S1: Temporal dependency of auxiliary variables (rectangles). The solid line is prescribed, the dashed line may be inferred from data.

# Constraints on Bayesian Network Structures


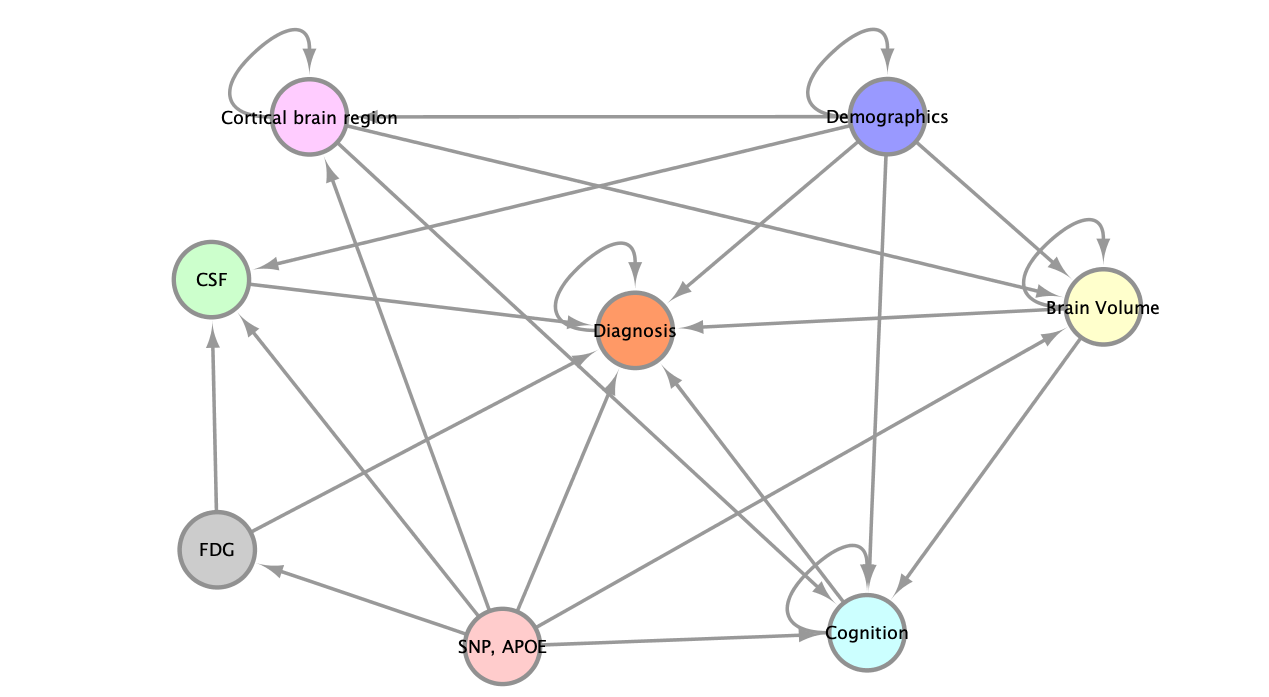


Figure S2: Potentially allowed edges between different variable groups in ADNI.


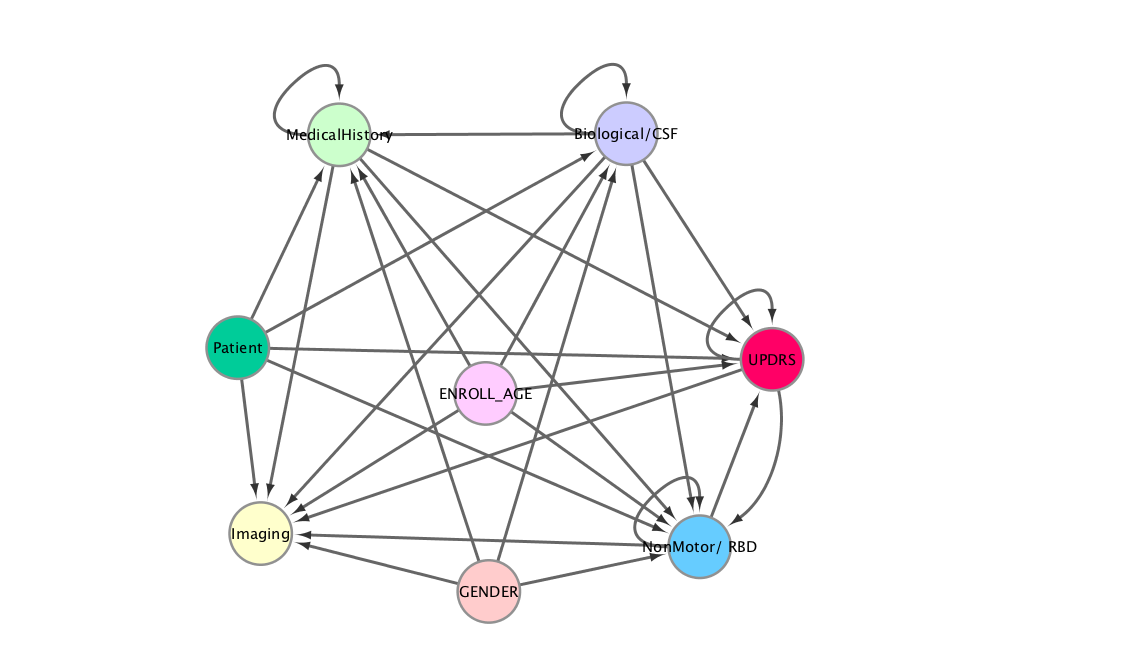


Figure S3: Potentially allowed edges between different variable groups in PPMI.

#

# Definition of Feature Groups

| **Group** | **Contained Features** | **#Bins** | **MSE of autoencoder** | **p.adjust (real vs simulated data)** |
| --- | --- | --- | --- | --- |
| brain.* | brain regions: Hippocampus, Entorhinal, Ventricles, Fusiform, Intracranial volume (ICV), Mid temporal lobe | Baseline: 4 Month 6: 12  Month 12: 4 Month 24: 2 | Baseline: 0.016  Month 6: 0.013  Month 12: 0.013  Month 24: 0.013 | Baseline - 1  Month 6 - 1  Month 12 - 1  Month 24 - 1 |
| Cog.* | cognition scores: MMSE, MOCA, CDRSB, ADAS11, ADAS13, RAVLT, FAQ | Baseline: 6 Month 6: 3  Month 12: 3  Month 24: 2 | Baseline: 0.022  Month 6: 0.013  Month 12: 0.016  Month 24: 0.017 | Baseline - 1  Month 6 - 0.2616  Month 12 - 0.4262  Month 24 - 0.1625 |
| CSF | ABETA, TAU, PTAU | Baseline: 2 | Baseline: 0.015 | Baseline - 1 |
| SNP.bl | APOE status + 110 SNPs | Baseline: 2 | Baseline: 0.06 | Baseline - 1 |
| brain68.bl | 68 cortical brain regions | Baseline: 2 | Baseline: 0.016 | Baseline - 1 |
| FDG | PET imaging diagnostics | Baseline: 4 | NA | FDG - 1 |
| Demographic features (treated separately) | Age, Gender, Education, Race, Ethnicity, Marital status | Age: 11 Gender: 2 Education: 16  Race: 4 Ethnicity: 3  Marital status: 4 | NA | Age - 1  Gender - 1  Education - 1  Race - 1  Ethnicity - 1  Marital status - 1 |
| DX.* | Diagnosis at baseline and subsequent time points | Baseline: 3  Month 6, 12, 24: 4 | NA | Baseline - 0.619  Month 6 - < 0.001  Month 12 - < 0.001  Month 24 - < 0.001 |

* Features considered as time dependent, NA (not applicable)

Table S3: feature groups defined for ADNI dataset, number of bins for each feature and mean square error (MSE) for each autoencoded feature. P-values correspond to a ${\chi^{2}}$-test (null hypothesis: virtual and real patient samples come from the same distribution). P-values were corrected for multiple testing using Bonferroni & Holm’s method. Note that p-values tend to become smaller the more samples are tested.

| **Annotation for clinical visit** | **Months** |
| --- | --- |
| bl | Baseline |
| m06 | Month 6 |
| m12 | Month 12 |
| m24 | Month 24 |

Table S4: Description of suffixes of variable names in ADNI.

| **Group** | **Contained Features** | **#Bins** | **MSE of autoencoder** | **p.adjust (real vs simulated data)** |
| --- | --- | --- | --- | --- |
| Patient_ENROL_AGE | Age at enrollment | 4 | NA | 1 |
| Patient_Simplified_Gender | Child bearing capacity | Male, Female | NA | 1 |
| Patient_Gender | Gender | Child bearing capacity- yes, no | NA | 1 |
| Patient demographic | Gender Ethnicity: Is subject Hispanic/Latino Identify self as Am Indian/Alaska Native Identify self as Asian Identify self as Black/African American Identify self as Hawaiian/Other Pacif Is Identify self as White Race not specified Origin population | 7 | NA | 1 |
| Patient PD history | Biological Mother Biological Mother with PD Biological Father Biological Father with PD Full Siblings Full Siblings with PD Half Siblings Half Siblings with PD Maternal Grandparents Maternal Grandparents with PD Paternal Grandparents Paternal Grandparents with PD Maternal Aunts and Uncles Maternal Aunts and Uncles with PD Paternal Aunts and Uncles Paternal Aunts and Uncles with PD How many children do you have How many children with PD  PD Family History Duration of the disease at enrollment  Handedness  Number of years of education | 7 | NA | 1 |
| UPDRS | UPDRS- Total Unified Parkinson's Disease Rating Scale score | Baseline-2,visit 1- 3,visit 2 -5, visit 3- 2, visit 4- 4, visit 5-2, visit 6-2,  visit 7- 2, visit 8- 1, visit 9- 2, visit 10- 2, visit 11- 6 | NA | Baseline - 1  visit 1 - 1  visit 2 - 1  visit 3 - 0.34  visit 4 - 0.01  visit 5 - 1  visit 6 - 1  visit 7 - 1  visit 8 - 1  visit 9 - 1  visit 10 - 1  visit 11 - 1 |
|  | UPDRS1-Non-motor experiences of daily living, | Baseline-3, visit 1- 3, visit 2 -4, visit 3- 5, visit 4- 4, visit 5-2, visit 6-4, visit 7- 2, visit 8- 2, visit 9- 3, visit 10- 4, visit 11- 3 | NA | Baseline - 1  visit 1 - 1  visit 2 - 1  visit 3 - 1  visit 4 - 1  visit 5 - 1  visit 6 - 1  visit 7 - 1  visit 8 - 1  visit 9 - 1  visit 10 - 1  visit 11 - 1 |
|  | UPDRS2-Motor experiences of daily living | Baseline-3, visit 1- 5, visit 2 -2, visit 3- 3, visit 4- 2, visit 5-6, visit 6-2, visit 7- 2, visit 8- 5, visit 9- 3, visit 10- 4, visit 11- 5 | NA | Baseline - 1  visit 1 - 1  visit 2 - 1  visit 3 - 1  visit 4 - 1  visit 5 - 1  visit 6 - 1  visit 7 - 1  visit 8 - 1  visit 9 - 1  visit 10 - 1  visit 11 - 1 |
|  | UPDRS3-Motor examination | Baseline-2, visit 1- 2, visit 2 -5, visit 3- 4, visit 4- 4, visit 5-2, visit 6-2, visit 7- 2, visit 8- 1, visit 9- 5, visit 10- 3, visit 11- 3 | NA | Baseline - 1  visit 1 - 1  visit 2 - 1  visit 3 - 0.51  visit 4 - 0.02  visit 5 - 1  visit 6 - 1  visit 7 - 1  visit 8 - 1  visit 9 - 1  visit 10 - 1  visit 11 - 1 |
| Medical history | WGTKG - Weight (in Kilograms), HTCM - Height (in Centimeters), TEMPC-Temperature (in Celsius), SYSSUP -Supine BP – systolic, DIASUP - Supine BP – diastolic, HRSUP-Supine heart rate, SYSSTND-Standing BP – systolic, DIASTND-Standing BP – diastolic, HRSTND- Standing heart rate | Baseline -2, visit 1 - 6, visit 2- 7, visit 3 - 7, visit 4- 4, visit 5 - 4, visit 6 - 4, visit 7 - 3, visit 8 - 4, visit 9- 5, visit 10 - 4, visit 11- 8 | Baseline: 0.019  Visit 1: 0.015  Visit 2: 0.013  Visit 3: 0.016  Visit 4: 0.018  Visit 5: 0.015  Visit 6: 0.018  Visit 7: 0.016  Visit 8: 0.019  Visit 9: 0.017  Visit 10: 0.016  Visit 11: 0.010 | Baseline - 1  visit 1 - 1  visit 2 - 1  visit 3 - 0.51  visit 4 - <0.01  visit 5 - 1  visit 6 - 1  visit 7 - 1  visit 8 - 1  visit 9 - 1  visit 10 - 1  visit 11 - 1 |
| Non-motor | DVT_TOTAL_RECALL-Derived-Total Recall T-Score DVS_LNS-Derived-LNS Scaled Score ESS- Epworth sleepiness scale  QUIP- Questionnaire for Impulsive-Compulsive Disorders in PD SCOPA-Scales for outcomes in Parkinson’s disease-autonomic STA-State Trait Anxiety Total Score | Baseline- 2, Visit 2- 6, visit 4- 4, visit 6- 5, visit 8- 4, visit 10- 3 | Baseline: 0.027  Visit 2: 0.018  Visit 4: 0.024  Visit 6: 0.023  Visit 8: 0.029  Visit 10: 0.023 | Baseline - 1  visit 2 - 1  visit 4 - 0.07  visit 6 - 1  visit 8 - 1  visit 10 - 1 |
| RBD | REM Sleep Behavior disorder (RBD) | Baseline- 2, Visit 2- 3, visit 4- 3, visit 6- 3, visit 8- 3, visit 10- 4 | NA | Baseline - 1  visit 2 - 1  visit 4 - 1  visit 6 - 1  visit 8 - 1  visit 10 - 1 |
| Cerebrospinal (CSF)* | Abeta 42 (pg/ml) | Baseline - 2, | NA | Baseline - 1 |
|  | CSF Alpha-synuclein (pg/ml) | Baseline -2, visit 2 - 3, visit4 -4, visit6 - 4,visit 8 - 2 | NA | Baseline - 1  visit 2 - 1  visit 4 - 0.52  visit 6 - 1  visit 8 - 1 |
|  | p-Tau181P (pg/ml) | Baseline - 3 | NA | Baseline - 1 |
|  | Total tau (pg/ml) | Baseline - 2 | NA | Baseline - 1 |
|  | t-tau/Abeta 1-42 | Baseline - 3 | NA | Baseline - 1 |
|  | p-tau/Abeta 1-42 | Baseline - 4 | NA | Baseline - 1 |
|  | p-tau/t-tau | Baseline - 3 | NA | Baseline - 1 |
| Biological | ALDH1A1 (rep 1),ALDH1A1 (rep 2), GAPDH (rep 1), GAPDH (rep 2), HSPA8 (rep 1), HSPA8 (rep 2), LAMB2 (rep 1), LAMB2 (rep 2), PGK1 (rep 1), PGK1 (rep 2), PSMC4 (rep 1), PSMC4 (rep 2), SKP1 (rep 1), SKP1 (rep 2), UBE2K (rep 1), UBE2K (rep 2). | Baseline - 4, visit 8 - 6 | Baseline: 0.011  Visit 8: 0.007 | Baseline - 1  visit 8 - 1 |
| Imaging | MRI results | 3 | NA | Baseline - 1 |

Table S5: Feature groups defined for PPMI dataset. *= treated as individual variables. For UPDRS “off medication scores” were used. P-values correspond to a ${\chi^{2}}$-test (null hypothesis: virtual and real patient samples come from the same distribution). P-values were corrected for multiple testing using Bonferroni & Holm’s method. Note that p-values tend to become smaller the more samples are tested.

| **Annotation for clinical visit** | **Months** |
| --- | --- |
| V00 | Baseline |
| V01 | Visit 01 (Month 3) |
| V02 | Visit 02 (Month 6) |
| V03 | Visit 03 (Month 9) |
| V04 | Visit 04 (Month 12) |
| V05 | Visit 05 (Month 18) |
| V06 | Visit 06 (Month 24) |
| V07 | Visit 07 (Month 30) |
| V08 | Visit 08 (Month 36) |
| V09 | Visit 09 (Month 42) |
| V10 | Visit 10 (Month 48) |
| V11 | Visit 11 (Month 54) |

Table S6: Description of suffix to variable names in PPMI.

#

# Autoencoder Training

Sparse autoencoders were trained for brain volumes, cognitive scores, csf features, snp features and cortical brain regions for ADNI data (Tables S3) and medical history, non-motor and biological features for PPMI data (Table S5). The loss function optimized by the autoencoder networks was the mean squared error loss (MSE).

Tuned hyper-parameters of autoencoder networks included the activation function (rectified linear unit or hyperbolic tangent), the input dropout ratio (0%, 5%, 20%, 50%), l2 penalty (10^-4^, …, 10^4^), and the network architecture. More specifically, we tested the following architectures:

- one hidden layer with one hidden unit
- two hidden layers: first layer with n / 2 units, second with one hidden unit
- three hidden layers: first layer with n/2 units, second with n/4 units and third with one hidden unit

For each combination of hyper-parameters a separate autoencoder training was performed for at most 500 epochs, but stopped earlier, if the MSE did not improve for 5 rounds. The best autoencoder model was selected according to the MSE criterion. We here relied on the h2o autoencoder implementation (<http://docs.h2o.ai/>).

Variable importances calculated according to the method described in the main paper can be found in the accompanying Excel sheets.

# Comparison of Different BN Structure Learning Algorithms

#
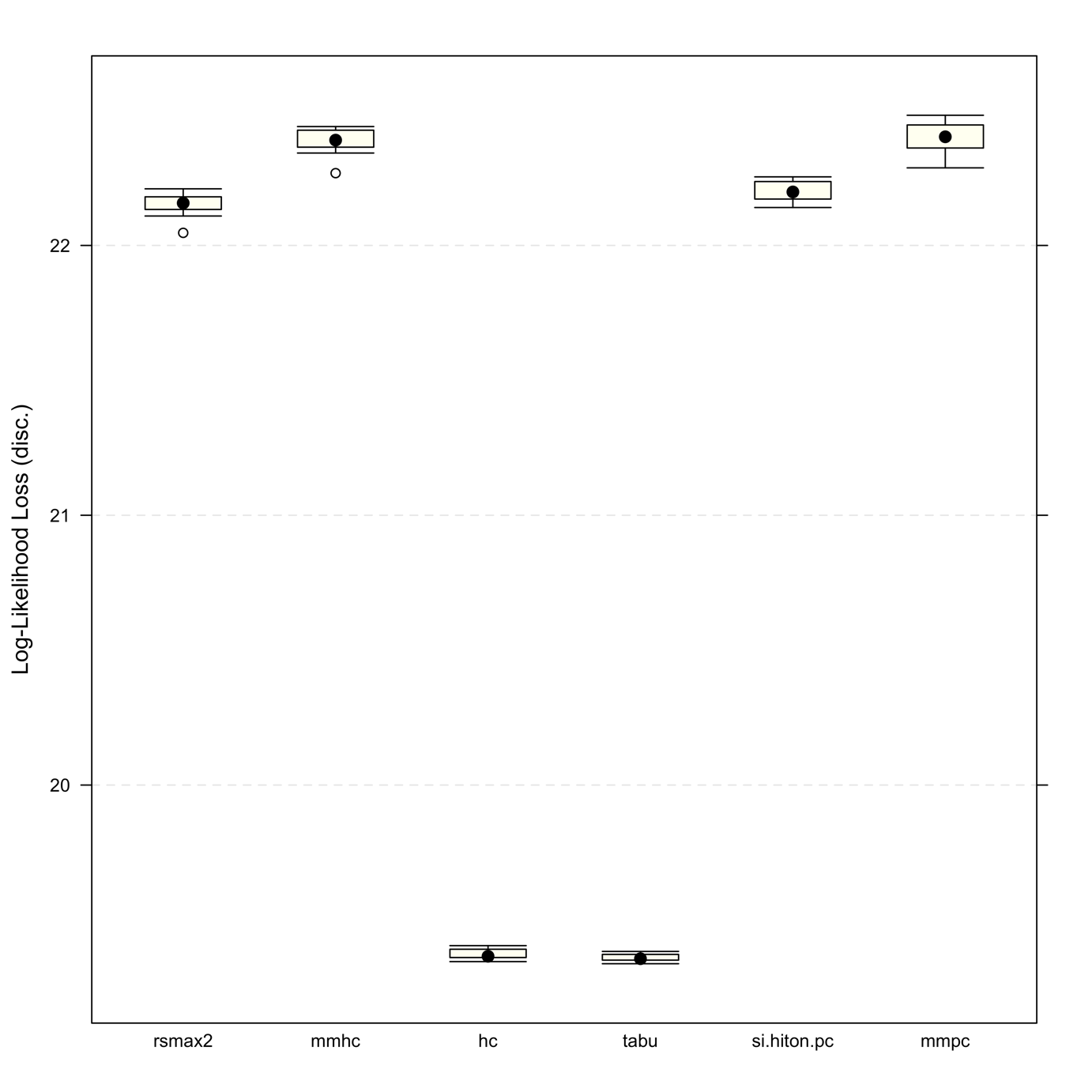


Figure S4: Comparison of different BN structure learning algorithms via 10-fold cross-validation for **ADNI** dataset. The y-axis depicts the negative log-likelihood of the test data.


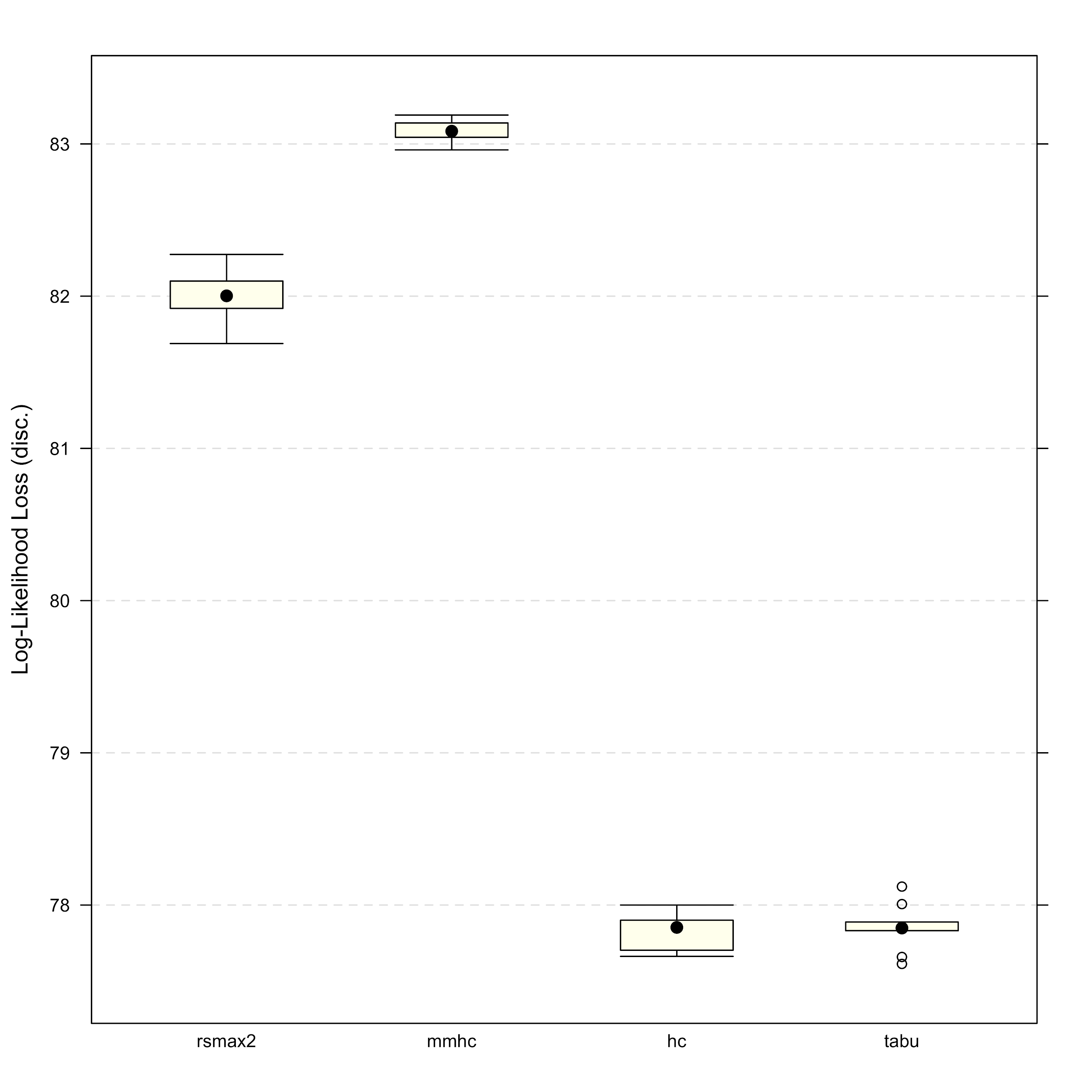


Figure S5: Comparison of different BN structure learning algorithms via 10-fold cross-validation for **PPMI** dataset. The y-axis depicts the negative log-likelihood of the test data. The two Markov Blanket learning algorithms (SI-HITON-PC, MMPC) are not shown, because their implementation in the bnlearn package resulted into an error message.

# Bayesian Network Structures


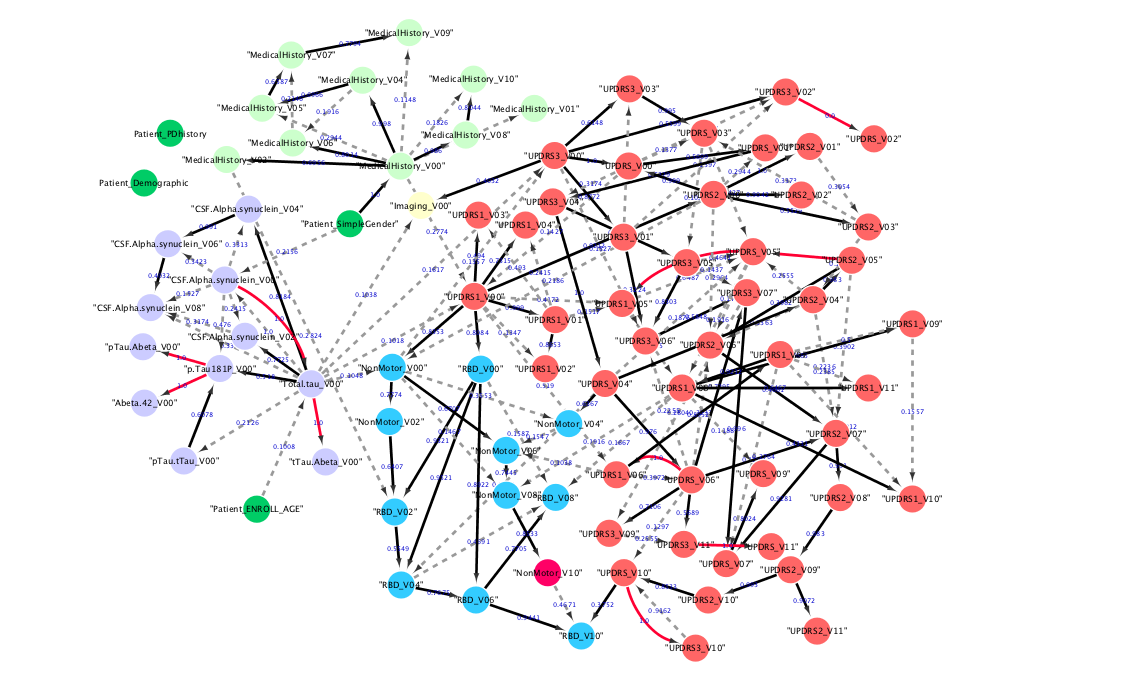


Figure S6: Variable dependencies identified in PPMI dataset in more than 100 / 1000 bootstrapped Bayesian Network reconstructions (dashed lines) and the final Bayesian Network (learned on the entire dataset, red lines), respectively. Solid edges indicate variable dependencies that are found commonly in bootstrapped Bayesian Network reconstruction and the final Bayesian Network topology. Auxiliary variables are not shown to simplify the representation.

# Comparison of Conservative Simulation Approach vs Direct Drawing from Bayesian Network

## ADNI
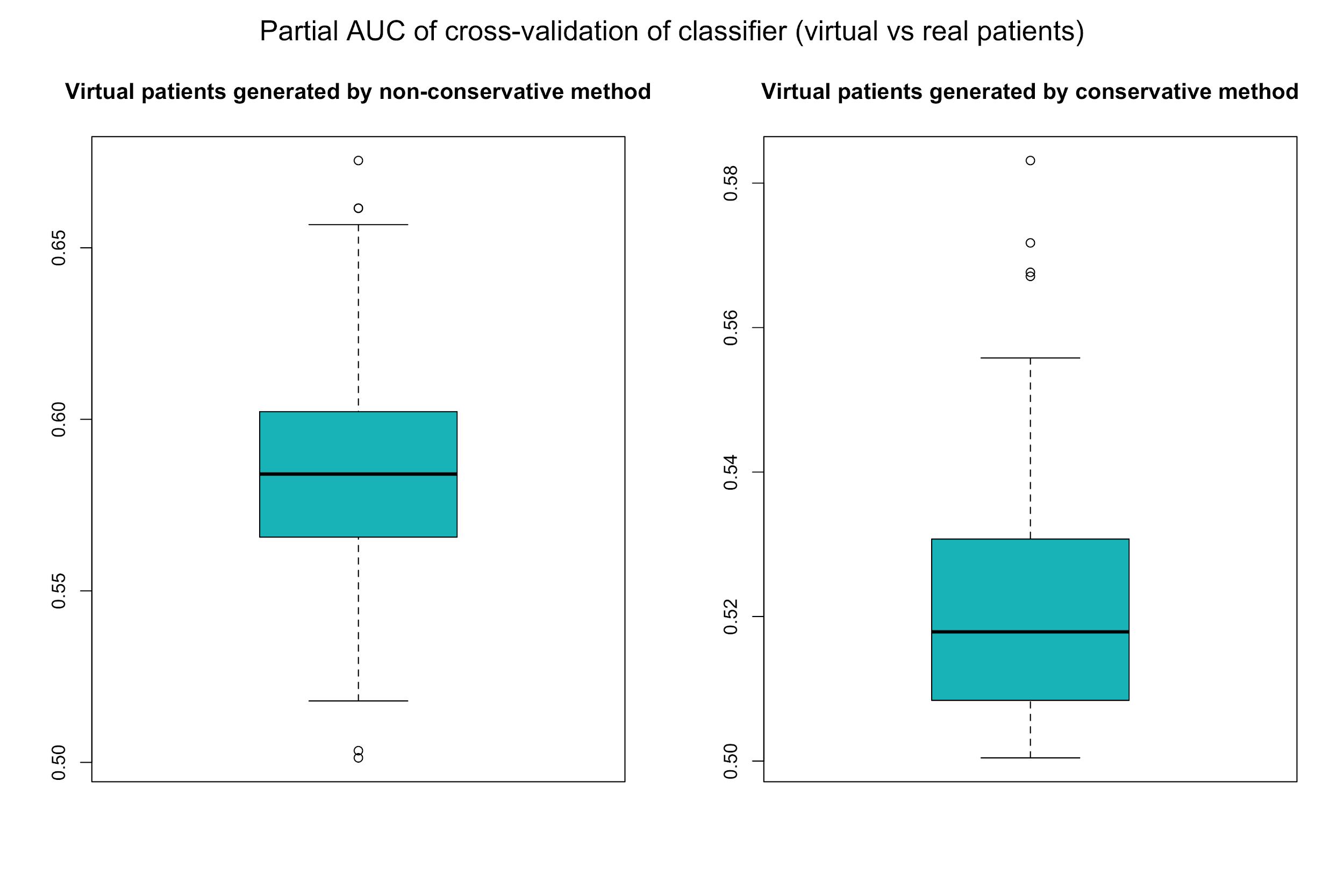


Figure S7: Performance of a Random Forest to correctly identify virtual subjects, measured via the partial area under ROC curve (pAUC) at a pre-specified detection rate of >=90 for real patients. The pAUC was assessed on test sets within 10 repeats of a 10-fold cross-validation procedure. Accordingly, boxplots show the distribution of the 10-fold cross-validated pAUC that was obtained from 10 repeats of the cross-validation procedure. The left plot shows the performance, if the VC is obtained by directly drawing from the BN. The right plot shows the performance when using our suggested conservative approach.

## PPMI


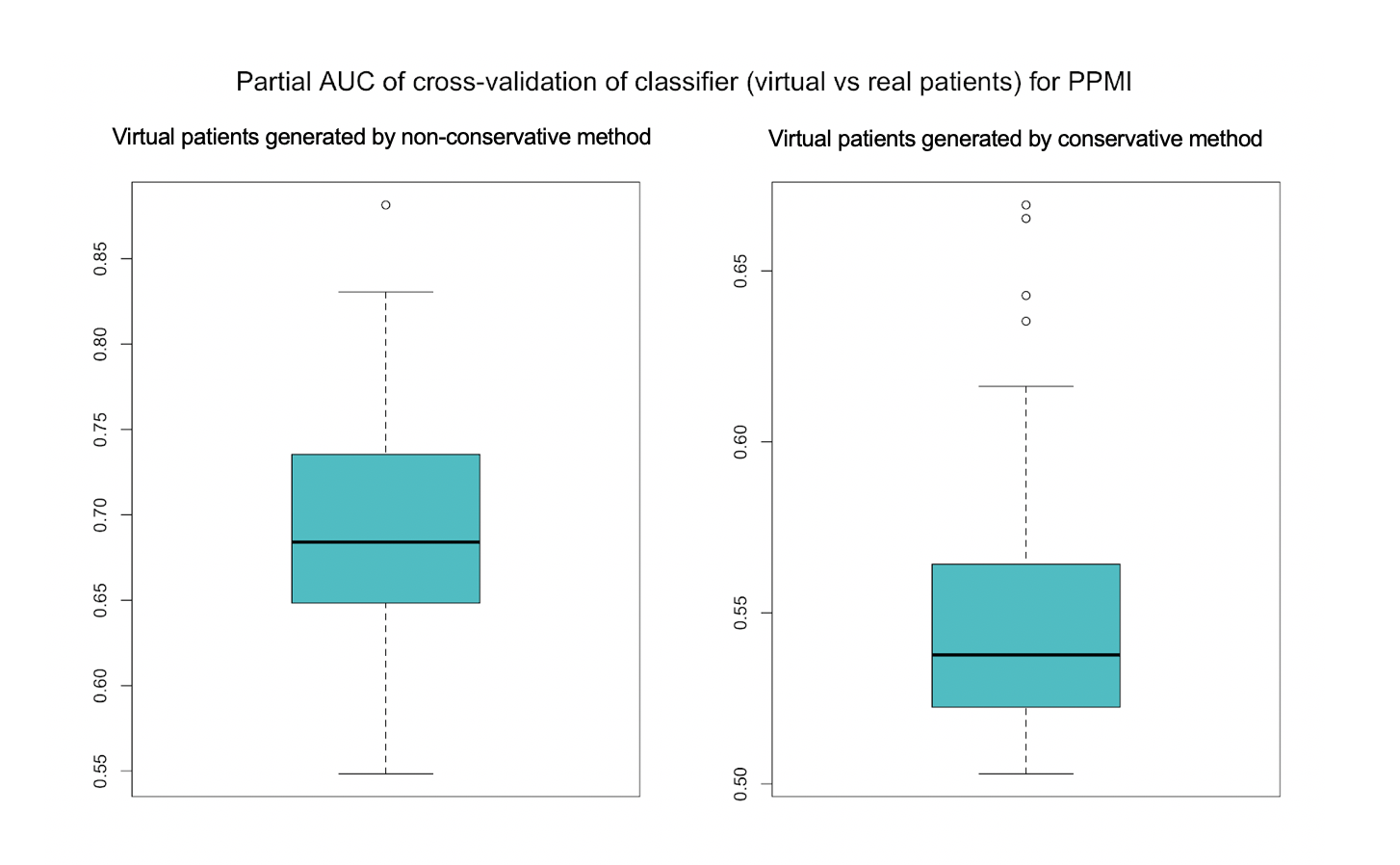


Figure S8: Performance of a Random Forest to correctly identify virtual subjects, measured via the partial area under ROC curve (pAUC) at a pre-specified detection rate of >=90 for real patients. The pAUC was assessed on test sets within 10 repeats of a 10-fold cross-validation procedure. Accordingly, boxplots show the distribution of the 10-fold cross-validated pAUC that was obtained from 10 repeats of the cross-validation procedure. The left plot shows the performance, if the VC is obtained by directly drawing from the BN. The right plot shows the performance when using our suggested conservative approach.

##

## Histogram plots of virtual and real patients


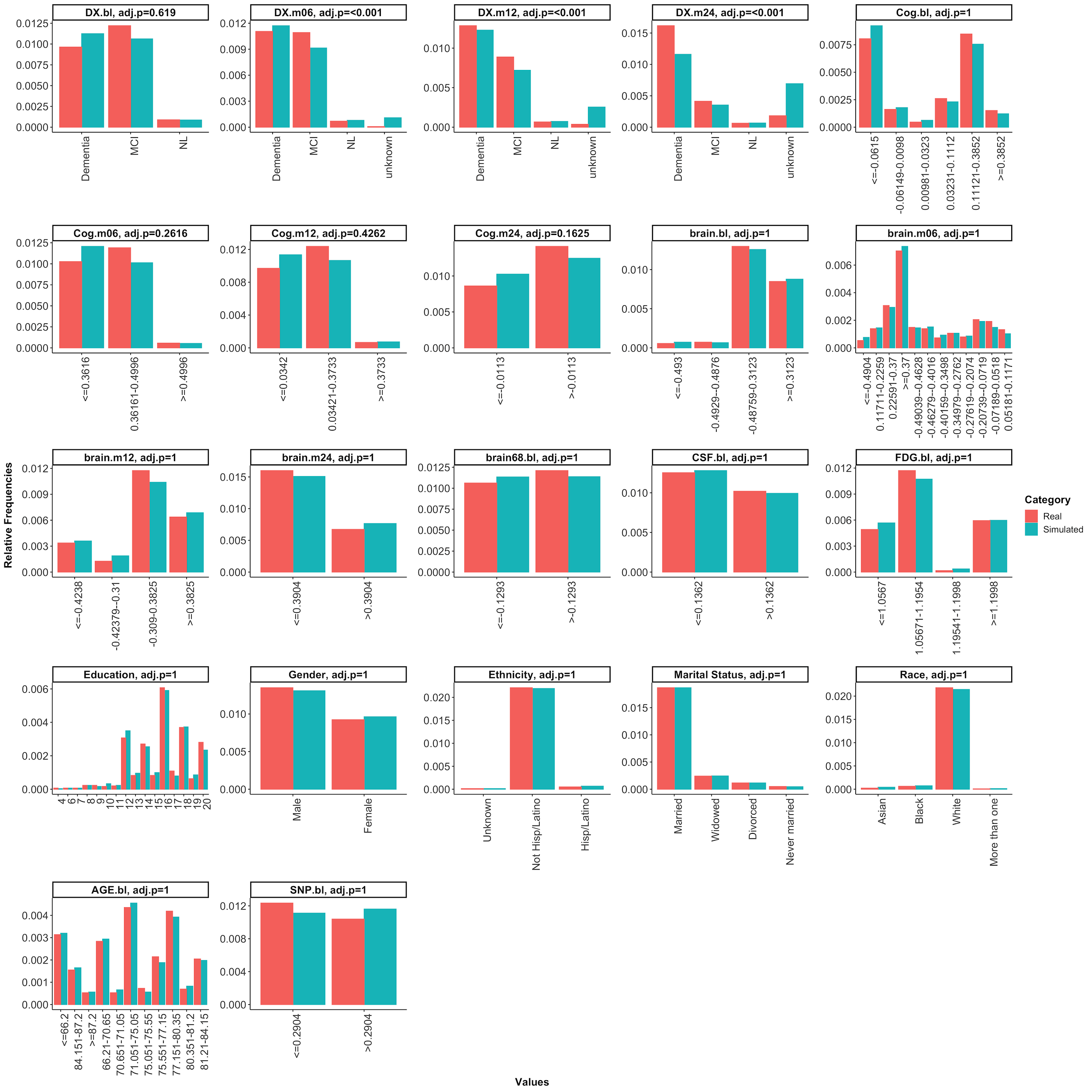


Figure S9: Histogram plots of individual variables in the original **ADNI** data (red) and in the VC (blue), adjusted p values displayed for each plot based on homogeneity test of each variable for real and simulated data


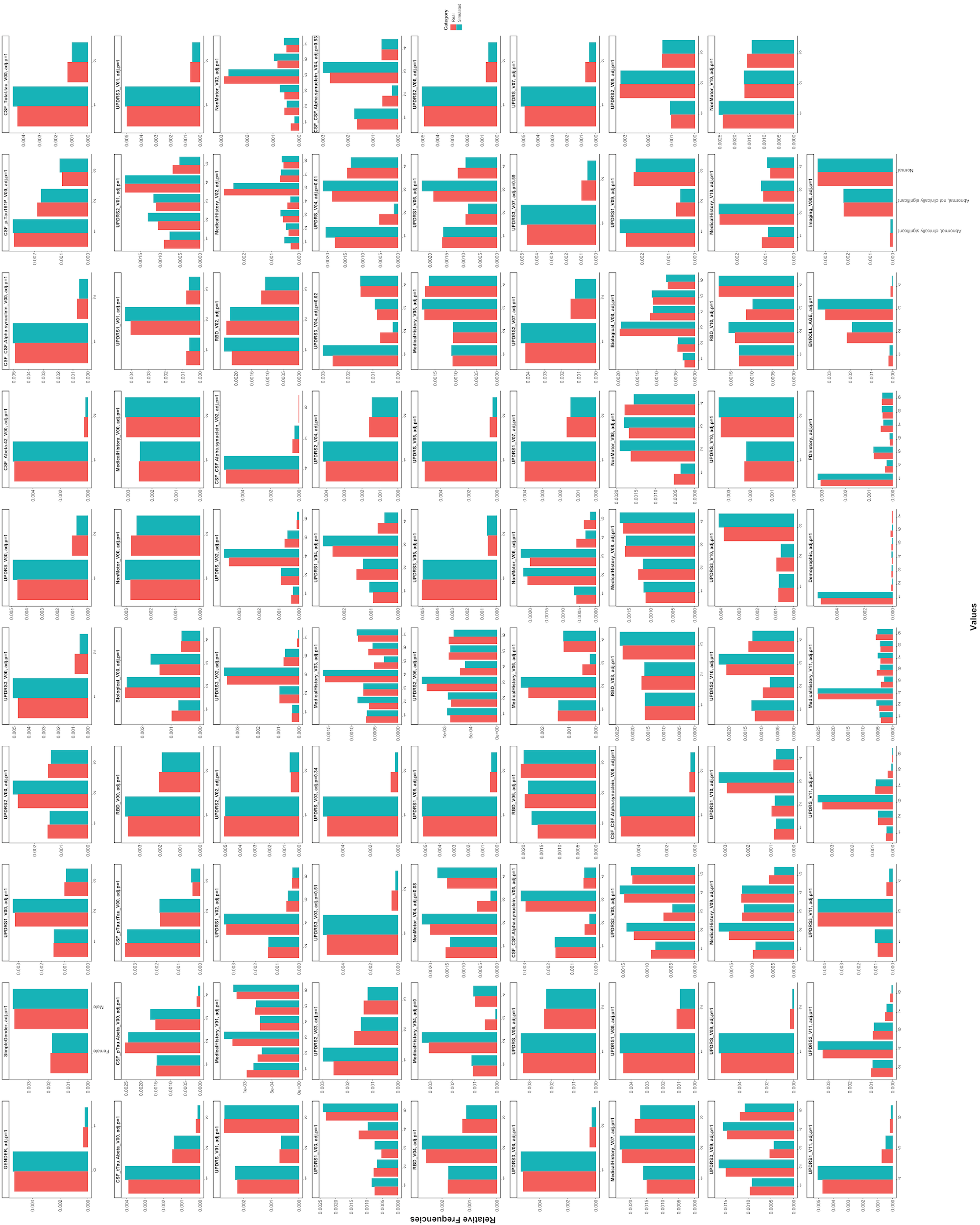


Figure S10: Histogram plots of individual variables in the original **PPMI** data (red) and in the VC (blue), adjusted p values displayed for each plot based on homogeneity test of each variable for real and simulated data

## Classifier trained on virtual patients generated from a Hybrid Gaussian BN

##

##
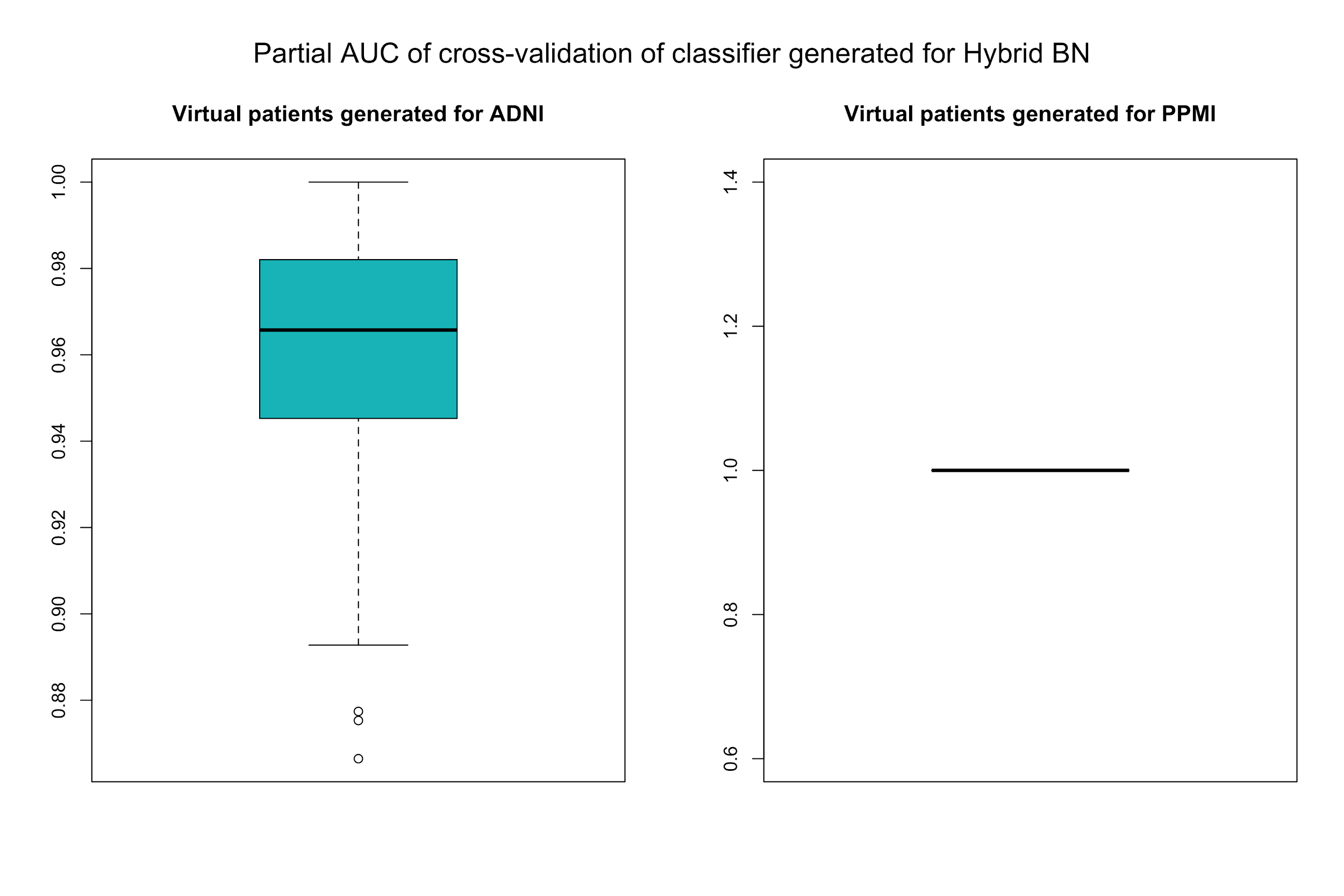


Figure S11: Drawing of virtual patients from hybrid Gaussian / discrete BN. The boxplots show the performance of a Random Forest to correctly identify virtual subjects, measured via the partial area under ROC curve (pAUC) at a pre-specified detection rate of >=90 for real patients. The pAUC was assessed on test sets within 10 repeats of a 10-fold cross-validation procedure. Accordingly, boxplots show the distribution of the 10-fold cross-validated pAUC that was obtained from 10 repeats of the cross-validation procedure. The left plot shows the performance for ADNI, and the right one for PPMI. Importantly, the boxplots are based on direct drawings of a VC from the hybrid BN. If the conservative approach suggested in the paper was chosen, all virtual patients were rejected (for both, ADNI and PPMI).
